# Cell-to-cell variation and specialization in sugar metabolism in clonal bacterial populations

**DOI:** 10.1371/journal.pgen.1007122

**Published:** 2017-12-18

**Authors:** Nela Nikolic, Frank Schreiber, Alma Dal Co, Daniel J. Kiviet, Tobias Bergmiller, Sten Littmann, Marcel M. M. Kuypers, Martin Ackermann

**Affiliations:** 1 Institute of Biogeochemistry and Pollutant Dynamics, Department of Environmental Systems Science, ETH Zurich, Zurich, Switzerland; 2 Department of Environmental Microbiology, Eawag, Duebendorf, Switzerland; 3 Institute of Science and Technology Austria (IST Austria), Klosterneuburg, Austria; 4 Division of Biodeterioration and Reference Organisms, Department of Materials and Environment, Federal Institute for Materials Research and Testing (BAM), Berlin, Germany; 5 Department of Biogeochemistry, Max Planck Institute for Marine Microbiology, Bremen, Germany; Indiana University, UNITED STATES

## Abstract

While we have good understanding of bacterial metabolism at the population level, we know little about the metabolic behavior of individual cells: do single cells in clonal populations sometimes specialize on different metabolic pathways? Such metabolic specialization could be driven by stochastic gene expression and could provide individual cells with growth benefits of specialization. We measured the degree of phenotypic specialization in two parallel metabolic pathways, the assimilation of glucose and arabinose. We grew *Escherichia coli* in chemostats, and used isotope-labeled sugars in combination with nanometer-scale secondary ion mass spectrometry and mathematical modeling to quantify sugar assimilation at the single-cell level. We found large variation in metabolic activities between single cells, both in absolute assimilation and in the degree to which individual cells specialize in the assimilation of different sugars. Analysis of transcriptional reporters indicated that this variation was at least partially based on cell-to-cell variation in gene expression. Metabolic differences between cells in clonal populations could potentially reduce metabolic incompatibilities between different pathways, and increase the rate at which parallel reactions can be performed.

## Author summary

This study addresses a fundamental question in bacterial metabolism: do all individuals in a clonal population express the same metabolic functions, or do individuals specialize on different metabolic functions and assimilate different substrates? Reports about stochastic gene expression in bacterial populations raise the possibility that transcriptional differences between individuals translate into different metabolic behaviors, but the prevalence and magnitude of such effects is currently not known. Here, we quantified the assimilation of two isotope-labeled sugars by single *Escherichia coli* cells using nanometer-scale secondary ion mass spectrometry, an analytical approach seldom used in systems biology. By comparing sugar assimilation and gene expression dynamics, we were able to differentiate the metabolic profiles of individual cells. We observed a previously hidden level of cell-to-cell variation in metabolism: cells differed both in the total amount of sugar they assimilated, as well as with respect to which of the two sugars they preferentially assimilated. Intriguingly, a cell’s preference in sugar assimilation was only partially based on specialization in gene expression. Taken together, this study is a step towards understanding the magnitude and the relevance of metabolic differences between genetically identical cells.

## Introduction

The aim of this study was to analyze metabolic phenotypes and quantify the assimilation of nutrients at the level of individual bacterial cells. The motivation was to contribute towards filling a major gap in our understanding of the metabolism of bacteria and other microorganisms. Many basic principles and underlying mechanisms of bacterial metabolism are well known [1]. We know, for a given culture environment, the metabolic pathways that are expressed and the fluxes of metabolites through these pathways [2, 3]. What we know little about, however, is whether *each individual cell* expresses all these metabolic pathways, or whether there are substantial and functionally relevant metabolic differences between cells.

The notion of phenotypic differences between genetically identical microbial cells has emerged as a concept in biology in the 1970s [4]. Research on phenotypic heterogeneity received renewed attention in the last two decades, with the rise of new and improved tools for single-cell analysis [5, 6, 7]. Quantitative analysis of gene expression at the single-cell level has led to the discovery of substantial levels of variation that are independent of genetic and environmental differences. One source of this variation is that many cellular processes are based on molecules that occur in small numbers per cell, and processes that occur at low rates; consequently, fluctuations in these small numbers or variations in these rates lead to substantial phenotypic variation between single cells, and within one cell over time [5, 7, 8, 9].

What is the evidence for phenotypic variation in metabolism, and how might such variation matter for the dynamics in microbial populations? Previous studies have found that intermediate concentrations of nutrients or other inducers of metabolic activity lead to variation in expression of metabolic genes and, generally, variation in metabolism [10, 11, 12]. Other experiments have found that cells differ in their growth rates during growth in constant environmental conditions [13], or during controlled switches from one growth condition to another [14, 15, 16]. Genome-wide screening for phenotypic variation showed that genes involved in carbon utilization, energy production and metabolism have higher levels of phenotypic variation than genes of most other functional classes in *E*. *coli* [17]. Variation in the expression of metabolic genes and its influence on bacterial growth and metabolic activity has been shown in model bacterial organisms and environmental isolates [12, 18]. In addition, metabolic diversity has also been reported between cells that belong to the same operational taxonomic unit (but are not necessarily genetically identical) from diverse environmental samples [19, 20, 21, 22].

Such variation in metabolism can potentially have functional consequences. On one side, phenotypic variation in metabolism can result in some individuals growing much slower than population average, and thus lowering the population growth rate. On the other hand, there are possible benefits. One possible benefit is that variation allows a genotype to cope with fluctuating environments; if individuals carrying the same genotype express different metabolic phenotypes, this increases the chance that at least some individuals will fortuitously express phenotypic traits that allow them to continue to grow and divide in case the environment shifts suddenly [12, 14, 23, 24, 25]. Another potential benefit is that if individual cells in a clonal population differ in their metabolic phenotypes and specialize on different subsets of metabolic reactions, they could potentially benefit from performing these processes faster or more efficiently. This would be expected if the metabolic specialization of single cells resolved incompatibilities and biochemical conflicts [26, 27, 28]. Phenotypic differentiation in metabolism could thus provide benefits of specialization to single cells while providing the clonal population as a whole with the benefits of being a generalist, namely the ability to consume a wide set of nutrients [26, 28, 29]. Whether individual cells show substantial metabolic variation in steady state conditions, and how this impacts the dynamics in microbial populations, is largely unknown.

We thus set out to measure metabolic activities of single bacterial cells. As a model system, we used *Escherichia coli* growing on a combination of the two carbon sources, glucose and arabinose, in chemostats. At high carbohydrate concentration, cells only utilize glucose, and the uptake and catabolism of arabinose is blocked through inducer exclusion, a mechanism of carbon catabolite repression [30]. Catabolite repression vanishes at low sugar concentrations, where carbohydrate availability limits the population sizes that can be attained [31, 32]. Our goal was to measure uptake and assimilation of both sugars at the level of single cells, to ask whether cells differ from each other in their total sugar uptake, and whether individual cells specialize on one of the two substrates.

Most established approaches in systems biology are not well suited to answer these questions. The common single-cell methods allow quantifying gene expression and phenotypic traits that can be assessed optically, such as cell growth, division, survival and differentiation, but they do not allow quantifying actual uptake and assimilation of nutrients. We used an alternative approach that has mostly been used for analyzing microorganisms in natural communities: labeling with stable isotope-labeled nutrients, and the subsequent detection of assimilated nutrients by means of nanometer-scale secondary ion mass spectrometry (NanoSIMS). This technique allows precise quantification of the isotope content of individual cells [33, 34, 35]. In parallel, we analyzed transcriptional reporters for genes involved in the uptake of glucose and arabinose to infer underlying molecular mechanisms of variation in assimilation.

## Results

### Heterogeneity in metabolic activity and growth rate, and carbon source specialization of clonal cells cultivated in nutrient-limited chemostats

Our main focus was on determining the rate at which individual bacterial cells consume and assimilate the two sugars that were available in our experiments, glucose and arabinose. Specifically, we wanted to know whether individual cells differ in the relative amount of each of the two sugars they assimilate, and whether they assimilate different total amounts of the two sugars combined. We worked with clonal populations of *E*. *coli* strains that were derived from the commonly used laboratory strain MG1655 [36]. Clonal populations were grown in mini-chemostats [2] in media containing arabinose and glucose as the sole externally added carbon sources. We ran a set of replicated chemostats under conditions where population size was limited by the carbon source, and another set where population size was limited by the nitrogen source and hence under conditions of carbon excess, while keeping the population size unchanged (see Materials and Methods, and S1 File, ‘Supplementary Methods’). As comparison, we also analyzed clonal populations growing in batch cultures (S1 Fig).

To analyze metabolic activity of single cells, we used sugars that were labeled with stable isotopes: we used arabinose in which 99% of all carbon atoms were the stable ^13^C isotope, and glucose in which 97% of all hydrogen atoms were ^2^H, the deuterium isotope. Assimilation of arabinose thus leads to the accumulation of ^13^C into the bacterial biomass, and assimilation of glucose leads to the accumulation of deuterium. We used nanometer-scale secondary ion mass spectrometry (NanoSIMS) to quantify isotope enrichment in biomass of bacterial cells. Due to its high resolution, NanoSIMS provides precise intracellular measurements of various stable isotopes [33, 34, 35].

We first performed control experiments in chemostats where bacterial strains were grown on only one of the two sugars (see S1 File, 'Maximal incorporation of the stable isotope-labeled sugars in chemostats'), and analyzed the isotopic labeling that they maximally achieved. Interestingly, these experiments showed that bacteria grown on ^13^C-arabinose for ten generations (during which more than 99% of the biomass is newly formed) reached a ^13^C labeling of about 70%, and thus contained about 30% of ^12^C (S1 Table). The unlabeled carbon assimilated during this experiment is most likely derived from assimilable organic carbon (AOC), which is a collective term describing the fraction of labile dissolved organic carbon molecules that is readily assimilated by microorganisms, resulting in their growth [37]. It usually consists of a broad range of low molecular weight organic molecules such as sugars, organic acids, and amino acids. In our chemostat setup, AOC could originate from culture containers, medium components, and laboratory air [37]; in control experiments where we grew *E*. *coli* in batch cultures without adding sugar, we found that AOC alone can support growth of approximately 10^6^ cells/ml (S2 Fig, ‘Bacterial growth on AOC’ in S1 File). AOC made a considerable fraction of the assimilated carbon because the sugar concentration supplemented in the medium was relatively low, i.e. 20 μM (see S1 File for further discussion: ‘AOC contamination in the chemostat setup’). Bacteria grown on ^2^H-glucose for ten generations reached a ^2^H labeling of about 14% (S1 Table). The rest of their hydrogen was composed of ^1^H, most likely derived both from AOC molecules as well as through hydrogen exchange with water [13]. These results allowed us to estimate the uptake of sugars from sugar mixtures, as will be discussed below.

**Fig 1:**
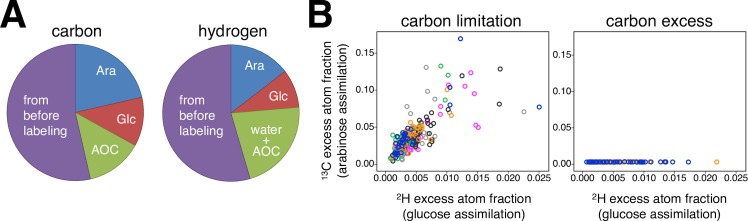
Isotopic composition of the biomass and heterogeneity in sugar assimilation. A Carbon and hydrogen from different sources were assimilated into the biomass of cells growing in carbon-limited chemostats. The chemostats were operated at a dilution rate of 0.15 h^-1^ corresponding to 25% of the maximum growth rate μ_MAX_ (see S2 File for μ_MAX_). Carbon was mostly ^12^C, except carbon originating from arabinose (depicted in blue) that was 99% ^13^C-labeled. Assimilable organic carbon (AOC, green) presented a considerable fraction of the total assimilated carbon. Hydrogen was mostly ^1^H, except hydrogen originating from glucose (red) that was 97% ^2^H-labeled. Hydrogen from water and AOC (green) presented a considerable fraction of the total assimilated hydrogen. Carbon and hydrogen assimilated before switching to media with labeled sugars remained the main contributors to the biomass (depicted in purple) (for calculations see S2 File, ‘Mathematical Model’). B Individual cells showed variation in the assimilation of glucose and arabinose. Each data point indicates excess atom fractions of a single cell in carbon-limited and nitrogen-limited (carbon-excess) chemostats, determined for ^2^H assimilation from labeled glucose as *X*^*E*^ (^2^H)_cell_, and for ^13^C assimilation from labeled arabinose as *X*^*E*^ (^13^C)_cell_. Population size in both chemostat setups was similar, about 2.4 x 10^6^ cells/ml. Populations growing in mixed-substrate carbon-limited chemostats assimilated both sugars, whereas populations growing in nitrogen-limited chemostats utilized solely glucose under a regime of catabolite repression. Replicate cultures are plotted in different colors.

We then grew bacteria in chemostats that contained both arabinose and glucose, to determine the metabolic behavior of single cells grown on mixed substrates. We supplemented the chemostats with unlabeled sugars until they reached steady state, and then switched to ^13^C-arabinose and ^2^H-glucose (S3 Fig). After an incubation period corresponding to 65% of the generation time we retrieved the cells from the chemostats and analyzed them with NanoSIMS. One expects that these cells contain isotopes of carbon and hydrogen from different sources. The two sugars provide a heavy isotope of C and H, respectively, but they also provide the light isotope of the other element; in addition, light isotopes stem from biomass assimilated before isotope labeling, and from AOC molecules as well as from water (in the case of hydrogen). To correct for these effects, we constructed a mathematical model that described the isotope composition of individual cells based on input from all these sources (see S2 File, ‘Mathematical Model’). The model also included that cells assimilate AOC and exchange hydrogen with water, but did not include variation between cells in these parameters, since we had no measurements that would allow estimating this for single cells. This model allowed estimating the sources of carbon and hydrogen for the cells grown in our chemostats; these estimates are depicted in Fig 1A. We will describe further below how we also used this model to estimate sugar assimilation for individual cells.

We then analyzed the ^13^C excess atom fraction as a marker for arabinose assimilation and the ^2^H excess atom fraction as a marker for glucose assimilation for each cell. In chemostats that were carbon-limited we observed variation in the excess atom fractions of ^13^C and ^2^H between cells, and also variation in the ratio of these two isotope fractions (Fig 1B). This indicates that bacteria consumed both sugars simultaneously, but that there was heterogeneity between cells in the total amount and the relative fraction of the two sugars assimilated. In contrast, cells from chemostats that were nitrogen-limited did not assimilate ^13^C from arabinose, as they grew under conditions of carbon excess. This observation is consistent with a large body of work on catabolite repression: many bacteria growing in the presence of high concentrations of glucose and a second sugar consume exclusively glucose [30].

While this analysis revealed variation in content of the two isotope markers between individual cells, it did not provide direct quantitative information about the rate at which the two sugars were assimilated; to obtain quantitative information, we needed to take into account the different sources of the two isotopes, as illustrated in Fig 1A. From the mathematical model described above we derived a proxy for the degree of assimilation specialization, which we refer to as *s* (see S2 File, ‘Mathematical Model’). A value of *s* = 0 indicates that cells are not specializing on either sugar, and consume and assimilate the sugars in the proportion they are available. *s* = -1 indicates that a cell only assimilates arabinose, and *s* = 1 indicates that a cell only assimilates glucose.

**Fig 2:**
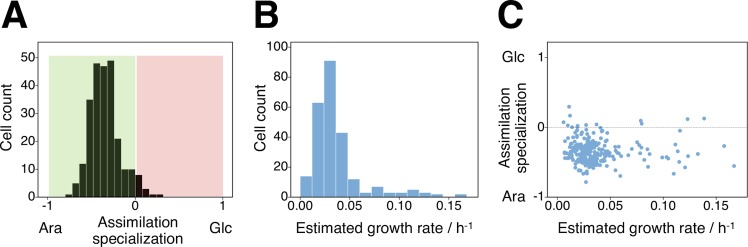
Degree of specialization and total growth rate in carbon-limited chemostats. A A cell’s preference for assimilating arabinose or glucose was calculated as a degree of specialization, *s* (see S2 File, ‘Mathematical Model’); *s* = 0 indicates no specialization in assimilation, *s* = -1 indicates that a cell only assimilates arabinose, and *s* = 1 indicates that a cell only assimilates glucose. The clonal population exhibited a continuous variation in specialization, and most cells assimilated both sugars. Still, 95% of the cells assimilated preferentially arabinose, and 5% of the cells assimilated preferentially glucose. B Single-cell growth rate was estimated from a cell’s growth on glucose, arabinose and AOC. C The variation in sugar preference (assimilation specialization *s*) was constant across cells with different single-cell growth rates.

We discovered large and unimodal variation in the degree of specialization between individual cells. While most cells did assimilate measurable amounts of both sugars, the ratio between glucose and arabinose that individual cells assimilated varied substantially (Fig 2A). This variation was unimodal in the sense that the degree of specialization of individual cells varied around one common mean value; we found no evidence of two distinct groups of cells, with one group specializing on glucose and the other on arabinose. For comparison, we conducted the same analysis with a strain of *E*. *coli* that was recently isolated from an environmental source [38], and found qualitatively similar results (Table 1, S4 and S5 Figs). This indicated that the large unimodal variation in the degree of specialization that we observed was independent of adaptation to laboratory conditions.

**Table 1:** Correlation analysis of the excess stable isotope-atom fractions and the expression levels of fluorescent gene reporters measured in carbon-limited chemostats.

	Spearman’s rho	p-value	N cells
**Sugar assimilation**			
Excess atom fractions ^2^H and ^13^C in NN114	0.730	< 2.2E-16	251
Excess atom fractions ^2^H and ^13^C in EAEC 55989	0.862	1.72E-15	49
**Transcriptional reporters**			
PptsG-*mCherry* and ParaE-*gfp*	0.642	< 2.2E-16	588
PrpsM-*gfp* and ParaE-*mCherry*	0.204	1.21E-11	1086
PrpsM-*gfp* and PptsG-*mCherry*	0.664	< 2.2E-16	793

The mathematical model did not only allow to estimate the *relative* assimilation of the two sugars, but also allowed estimating the total sugar consumption and assimilation of each cell, and thus the increase of its biomass through cellular growth during the incubation period with ^13^C-arabinose and ^2^H-glucose. This analysis again revealed large variation in growth rates between individual cells growing in carbon-limited chemostats (Figs 2B and S6). Two estimates illustrate the magnitude of this variation: the growth rate of the 20% most active cells exceeded the growth rate of the 20% least active cells by the factor of 5.5. And if all cells would grow as fast as the most active 20%, the population would grow 2.14 times faster. These results reveal large variation between cells in their total metabolic activity and growth, similar to what has recently been reported by [13].

Of note, variation in growth rate was more than three times higher in chemostat cultures than in batch cultures (S7 Fig; for the analysis of isotope enrichment in batch cultures see S2 File, ‘Mathematical Model’). This suggests that restricted growth in carbon- or nitrogen-limited chemostats promotes heterogeneity in the utilization of carbon sources, which translates into growth rate heterogeneity. We see this result as an interesting contrast to the conventional perspective that cells growing in chemostats are in physiological steady state [1].

The next question we addressed was whether the degree of specialization on glucose versus arabinose depended on the rate at which single cells grew. Did all cells show large variation in specialization, independently of how fast they grew? To address this question, we analyzed the proxy for assimilation specialization, *s*, as a function of the single-cell growth rate. This analysis revealed that preference for sugar assimilation did not depend on the single-cell growth rate (Fig 2C), and that the large variation in specialization that we observed was a robust pattern across cells with different growth rates.

Interestingly, we observed that most cells assimilated more carbon from arabinose than from glucose (Fig 2A). This does not mean that they *consumed* more arabinose, since both glucose and arabinose are expected to be depleted in such carbon-limited chemostats [31, 32]. Rather, it is possible that glucose was primarily used for energy production (with its carbon atoms not being assimilated into biomass) and arabinose primarily as a cellular carbon source, similar to what has been found in a recent study that investigated simultaneous utilization of methanol and succinate by *Methylobacterium extorquens* [39].

Overall, these direct measurements of metabolic activity based on stable isotope-labeled nutrients revealed large variation in total assimilation between single cells, as well as in the relative proportion of the two sugars assimilated. This raises the question about the cellular basis of this variation in metabolism. Specifically, we asked whether the variation in metabolic activity was mirrored in the expression of genes involved in sugar uptake and assimilation, and was potentially caused by variation in gene expression between cells. To this end we used fluorescent reporters to analyze transcription in single cells.

### Phenotypic variation in the expression of fluorescent reporters for sugar transporters in nutrient-limited chemostats

To investigate transcription of sugar uptake genes, we worked with strains that carried fluorescent transcriptional reporters for genes involved in the uptake of glucose and arabinose. Glucose can be taken up by at least five different systems in *Escherichia coli*, including the three main glucose transporters PtsG/Crr, ManXYZ and MglBAC [40, 41, 42]. Furthermore, *E*. *coli* has two arabinose transport systems, AraE and AraFGH [43, 44]. We tested transcriptional reporters for these genes [45] and found that their expression patterns varied depending on the availability and concentration of carbon sources (S8 and S9 Figs). Moreover, bacterial growth rate, i.e. dilution rate in chemostats, also affected expression patterns of transcriptional reporters (S10A Fig). From this set of reporters, we selected two genes for more detailed analysis, namely the glucose phosphotransferase system PtsG and the arabinose-proton symporter AraE; these genes showed a broad dynamic range of expression when tested in different conditions (S10 Fig), and previous studies had reported that these two genes have a high degree of phenotypic variation in clonal populations [17, 46]. To analyze the variation and covariation in the expression of these two genes in single cells, we constructed a strain in which two different fluorescent proteins served as transcriptional reporters for the two genes; this strain carried ParaE-*gfp* and PptsG-*mCherry* on its chromosome. We used fluorescence microscopy to quantify the levels of GFP and mCherry in individual cells.

**Fig 3:**
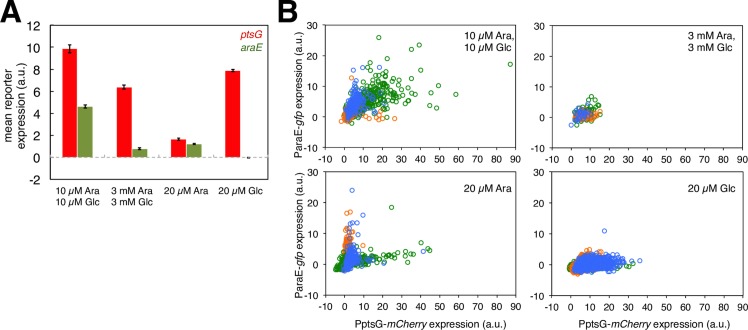
Expression of fluorescent transcriptional reporters for sugar transporters in chemostats. A Mean fluorescence levels of PptsG-*mCherry* and ParaE-*gfp* were measured for the MG1655-derived strain NN114. The data indicate low levels of *araE* expression in the absence of arabinose (glucose-limited chemostats) or in the presence of high glucose concentrations (carbon-excess nitrogen-limited chemostats). The error bars depict standard errors of the mean. B Each data point indicates fluorescence of a single cell harboring the PptsG-*mCherry* and ParaE-*gfp* reporters. Three replicate cultures are plotted in different colors. Generally, the distributions of expression of PptsG-*mCherry* and ParaE-*gfp* were significantly different across different conditions (Kolmogorov-Smirnov test: p-values< 0.001 and Mann-Whitney U test: p-values< 0.001 for pairwise comparisons of carbon-limited mixed-carbon conditions with carbon-excess mixed-carbon conditions, glucose-limited and arabinose-limited conditions for expression of both reporters), except for only slight differences in the mean PptsG-*mCherry* expression level between carbon-limited mixed-carbon and glucose-limited chemostats (Mann-Whitney U test: p-value< 0.05).

We then analyzed the expression of these transcriptional reporters both by comparing population means across different carbon source conditions as well as by comparing individual cells within a given condition. We found consistent changes in mean expression between chemostat conditions (Fig 3A): the promoter of *ptsG* was generally more active when bacteria were grown in the presence of glucose, while the promoter of *araE* was active when arabinose was present and not when only glucose was present. When analyzing the signal of the transcriptional reporter at the level of single cells (Figs 3B and S11), we found large variation in reporter expression levels between cells. Again, and importantly, this variation was unimodal around the population mean. We found no evidence for the emergence of two discrete groups of cells that each would specialize on the expression of genes to take up one of the two sugars (of note, the apparent formation of discrete groups with different gene expression phenotypes in cultures grown on 20 μM arabinose in Fig 3B is a consequence of differences in gene expression patterns between different replicate populations, rather than caused by the co-existence of differently specialized cells within the same replicate population).

The analysis of transcriptional reporters of single cells growing on mixtures of arabinose and glucose revealed a positive association between *araE* expression and *ptsG* expression (Fig 3B, Table 1). This observation is in line with the notion of substantial ‘global noise’ in gene expression [8]. Global noise manifests as variation in overall gene expression levels between individual cells, and can arise from differences between cells in the concentration of cellular components that modulate rates of transcription and translation. One possible source of global phenotypic noise is variation between cells in growth rates. Many bacterial promoters show an increase in activity with increasing growth rates [47, 48, 49]. One would thus expect that even among genetically identical cells growing under homogeneous conditions there might be an association between single-cell growth rates and overall transcriptional activity. As a first approach to address this question, we tested for an association between the expression levels of the reporters for the sugar transporters *ptsG* and *araE* and the ribosomal gene *rpsM*, which encodes the ribosomal protein S13. The rationale of this experiment was to use the expression of ribosomal proteins as a proxy for growth rate [47, 50]. This analysis revealed a significant correlation between the expression of reporters for sugar transporters and for *rpsM* (Table 1), in line with the notion that the activities across different types of promoters within a given cell were positively correlated, and potentially also correlated with the cell’s growth rate.

### Linking specialization in gene expression to specialization in metabolism

The observation of large degrees of cell-to-cell variation both in metabolic activity and in the expression of sugar uptake genes raised the question about how these two types of cellular processes were linked: was the relative specialization of a cell for glucose or arabinose mirrored in the expression levels of genes involved in uptake of glucose and arabinose measured at the same time? Such a pattern would indicate a direct link between a cell’s expression level of *ptsG* and *araE* and its uptake and assimilation of glucose and arabinose, respectively.

**Fig 4:**
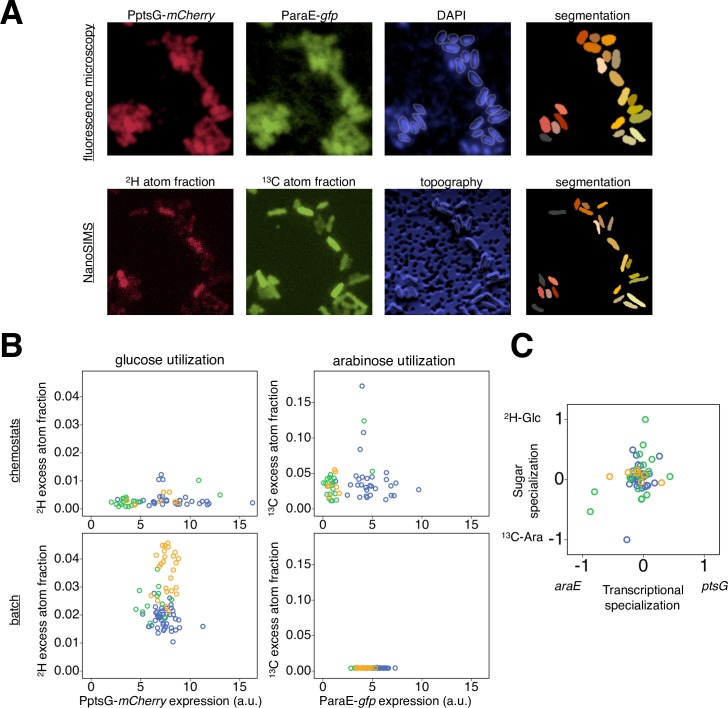
Combining fluorescence microscopy and NanoSIMS. A The cells were grown in well-mixed liquid cultures–batch or chemostats, fixed, and then deposited to the filters for NanoSIMS analysis. Therefore, cells that are close to each other on the filter were not close during their growth. For individual cells, we measured expression of ParaE-*gfp*, expression of PptsG-*mCherry*, ^13^C atom fraction and ^2^H atom fraction in the same area of interest. We analyzed only those cells that could be unambiguously defined and linked between raw NanoSIMS and fluorescence microscopy images (colored cells; other cells are depicted in grey). B Single-cell measurements of three independent replicate cultures (depicted in three different colors) grown in carbon-limited chemostats (10 μM of each labeled sugar) and carbon-excess batch cultures (1.5 mM of each labeled sugar) were plotted. There was no significant correlation between ^2^H excess atom fraction *X* ^*E*^(^2^H)_cell_ and PptsG-*mCherry* expression regarding glucose utilization (Spearman's rho = 0.120, p = 0.373, N = 57 cells in chemostats; rho = 0.063, p = 0.579, N = 81 cells in batch), nor between ^13^C excess atom fraction *X*^*E*^ (^13^C)_cell_ and ParaE-*gfp* expression regarding arabinose utilization (rho = 0.031, p = 0.821 in chemostats; rho = -0.197, p = 0.078 in batch) in neither of the two tested conditions. C We determined transcriptional specialization (PptsG-*mCherry* signal relative to ParaE-*gfp* signal) and sugar specialization (^2^H excess atom fraction relative to ^13^C excess atom fraction) for cells grown in carbon-limited chemostats, as defined in the Methods section ‘Analysis of filters with fluorescence microscopy and NanoSIMS’. Cells that have higher PptsG-*mCherry* signal relative to the ParaE-*gfp* signal also have higher ^2^H excess atom fraction relative to the ^13^C excess atom fraction, and *vice versa*. In other words, cells that specialize more on the *ptsG* transcription tend to have a stronger specialization on glucose assimilation. This observation is based on a positive parametric correlation between transcriptional specialization and sugar specialization (Pearson's r = 0.304, p = 0.021, N = 57 cells from 3 replicates depicted in different colors; non-parametric correlation analysis is not significant).

To address this question, we used an experimental approach to link the analyses of gene expression and sugar assimilation: we used fluorescence microscopy to analyze gene expression patterns in clonal groups of bacteria, and then used NanoSIMS on the same group of bacteria to analyze substrate assimilation at the single-cell level. For each cell, we therefore acquired four pieces of quantitative information: expression of ParaE-*gfp*, expression of PptsG-*mCherry*, assimilation of ^13^C-arabinose and assimilation of ^2^H-glucose (Fig 4A). We then tested for a statistical association between each cell’s expression level of *ptsG* and its glucose assimilation, and of its *araE* expression and arabinose assimilation. We found no significant direct correlation between the transcriptional signal of the transporter gene and the assimilation of the corresponding sugar, neither in carbon-limited chemostats nor in carbon-excess batch cultures (Fig 4B).

However, we found evidence that each cell’s *specialization* in gene expression was linked to its *specialization* in sugar assimilation in carbon-limited chemostats (Fig 4C). Specifically, we observed that cells that transcribe more *ptsG* than *araE* also tend to assimilate more glucose than arabinose. This analysis is based on two proxies of ‘specialization’ that we derived. First, we calculated the ‘transcriptional specialization’ of individual cells; we standardized reporter gene expression levels across different measurements and transformed them to obtain a proxy for the degree of specialization in gene expression. Second, we calculated the ‘sugar specialization’ of these cells in the same way, by standardizing and transforming isotope enrichment levels to obtain a proxy for the degree of specialization in sugar assimilation (see Methods, ‘Analysis of filters with fluorescence microscopy and NanoSIMS‘). We found that cells that have higher PptsG-*mCherry* signal than ParaE-*gfp* signal also have higher ^2^H excess atom fraction compared to ^13^C excess atom fraction. In other words, a cell that specializes on *ptsG* expression rather than *araE* expression tends to specialize on glucose assimilation rather than arabinose assimilation, and *vice versa* (Fig 4C). This indicates that in environments containing different sugars, the cell’s preference in sugar uptake and assimilation is at least partially based on specialization in transcriptional activity.

## Discussion

Our first main finding is that clonal cells in carbon-limited chemostats growing in the presence of two sugars show variation in cellular carbon assimilation rates (Fig 1B), and therefore presumably variation in the rate at which single cells grow (Fig 2B). This result is in line with results from recent chemostat experiments that were also based on measuring the incorporation of stable isotopes [13] or based on measuring fluorescent sugar analogues [51], and thus supports the growing notion that well-mixed chemostat cultivation can promote cell-to-cell heterogeneity [51]. There is one potential additional aspect of metabolic heterogeneity that our approach did not allow to quantify: it is possible that cells growing in carbon-limited chemostats could also differ in the assimilation of AOC.

Cell-to-cell variation in assimilation and growth rate can also arise in situations where cells grow on only one substrate. Our experiments in which clonal populations were grown in nitrogen-limited chemostats with excess of carbon sources indicates that even though carbon catabolite repression forces cells to utilize solely glucose, cells differ in the amount of assimilated glucose, which then manifests as the variation in single-cell growth rate (Fig 1B). It has been argued that limitation in other nutrients affects the degree of glucose utilization [52]. In our experiments, it is possible that limitation in nitrogen poses restrictions on glucose metabolism that do not affect every cell equally.

We also found that the variation in growth rate is significantly higher in chemostat cultures than in batch cultures. It is plausible that growth restriction imposed by the chemostat dilution rate generates an additional layer of heterogeneity that could manifest as variation in the rate at which single cells grow. Similar findings have been reported in recent studies that revealed the highest cell-to-cell variation in growth rates in the slowest growing cultures [13, 18]. The dilution rate can significantly affect gene expression patterns (S10A Fig), as well as intracellular fluxes [2] and the physiology of chemostat populations [31, 32], and it seems conceivable that these effects could contribute to variation in growth rates.

Our second main finding is that cells that grow in the presence of two sugars do not all assimilate similar amounts of these two sugars; rather, there is strong variation between single cells in their specialization on glucose versus arabinose (Fig 2A). While we did not find two *metabolically distinct groups* (but rather a continuous variation between cells in their sugar specialization), this result gives credence to the idea that there is substantial variability in the metabolic reactions performed by cells in clonal populations. As discussed in the Introduction, this notion of individual cells differing in the sets of reactions they perform raises the question whether such specialization can reduce biochemical conflicts within cells and increase the rate at which single cells grow.

Different cellular mechanisms could cause such cell-to-cell variation in sugar assimilation. Metabolic differences between genetically identical cells are often attributed to cell-to-cell variation in the transcription and translation of the relevant genes [7, 9, 28]. Alternatively, and not mutually exclusively, metabolic variation could potentially reflect differences in distribution of nutrient transporters due to for instance molecular sieving effect [53] or inner membrane protein crowding [54], and asymmetric partitioning [55] of the nutrient transporters as well as cytoplasmic macromolecules for the storage of energy or carbon [56] during cell division. Here, we asked whether we can explain part of the cell-to-cell variation that we observed in our NanoSIMS analysis by variation in the transcriptional activity between cells. Such an analysis is interesting because the combination of fluorescence reporters and NanoSIMS allows to directly test for a link between transcriptional activity and actual metabolism–something that is usually difficult to do.

Interestingly, when focusing on the assimilation of individual sugars from the mixtures of arabinose and glucose, we found no direct correlation between transcriptional initiation of the genes encoding the sugar transporters and assimilation of the respective sugars (Fig 4B). A first potential reason for this lack of a direct correlation is the redundancy of sugar transporters. *E*. *coli* has five different transporters to take up glucose [40, 41] and two different transport systems for arabinose [43, 44]. The expression of these systems is adjusted according to the carbon source concentrations in the environment and bacterial growth rates. It is thus plausible that individual cells employ different transporters to a different extent (S9 Fig) [46]. One experimental approach to investigate the role of transporter redundancy in our system would be to delete all genes encoding alternative sugar transporters from the genome. However, deleting these genes is expected to cause pleiotropic effects on several phenotypic traits including carbon utilization, as recently investigated in more details at the level of bulk populations [42], and is thus not easily feasible.

A second potential reason for the discrepancies between the transcriptional signal and a cell's actual activity could be caused by post-transcriptional regulation, including regulation of the mRNA stability, regulation of the production and the stability of the protein, and through post-translational modifications as for instance acetylation [57]. Indeed, it has been shown that *ptsG* expression is post-transcriptionally regulated by the small RNA SgrS [41] (post-transcriptional regulation of *araE* expression has not been reported [44]). Furthermore, even if transcriptional variation *does* translate into differences in enzyme concentrations and activities between cells, this does not necessarily lead to metabolic differences [3, 58, 59]. Almost all enzymes that are part of the central metabolic pathway are overabundant in the cell’s cytoplasm [60], so that enzyme variation should have little effect on the flux through a pathway (however, see [18] for a contrary example for how variation in the expression of an enzyme can directly affect bacterial growth).

Despite this lack of a direct correlation between transcription of a particular transporter gene and the assimilation of the sugar taken up by the corresponding transporter (Fig 4B), we found evidence for a different link between transcription and assimilation (Fig 4C). As described in the Results, we observed that cells that specialize on transcribing *ptsG* (encoding the glucose transporter) over transcribing *araE* (encoding the arabinose transporter) also tended to specialize on the assimilation of glucose over arabinose (Fig 4C). How is this consistent with the fact that we did not find a direct correlation between transcription of a transporter-encoding gene and the assimilation of the sugar taken up by the respective transporter? One possible explanation for this apparent inconsistency is the high level of variation in *total* carbon assimilation between cells that we observed (Fig 1B). If this variation in total assimilation is unrelated to transcription of *ptsG* and *araE*, then it is expected to obscure any direct correlation between transcription of a transporter-encoding gene and assimilation of the corresponding sugar. Determining the degree to which individual cells *specialize* on one sugar versus the other, as we did, removes this effect of variation in the total assimilation–and this analysis might therefore reveal the link between transcription and assimilation that manifests in Fig 4C. In summary, metabolic variation between cells might fundamentally be determined–at least in part–by what sets of genes these cells transcribe: cells that specialize more on the transcription of a gene encoding a certain sugar uptake system also show stronger specialization in the assimilation of this sugar.

From a broader perspective, we see an interesting connection between cell-to-cell variation in clonal populations and the functioning of genetically diverse microbial communities. One major question about diverse microbial communities is how different types of microorganisms complement each other to together form a distributed metabolic network. It is interesting to ask whether such a distributed metabolism can also arise–to a lesser extent–within clonal populations. If genetically identical cells in clonal populations perform different sets of metabolic processes, this could potentially alleviate metabolic incompatibilities and allow individuals to reap the benefits of metabolic specialization. One example of a distributed metabolism in microbial communities is a process called cross-feeding: one microorganism partially degrades a primary resource and generates a metabolic intermediate that is excreted and used as a resource by a second microorganism. Although this process has been mostly implicated to occur between genetically different populations [61, 62], there is some evidence of phenotypic cross-feeding. Transcriptional analysis suggests that under certain conditions clonal populations of *E*. *coli* [46], *Pseudomonas putida* [63], and potentially *Rhodopseudomonas palustris* [64, 65] might differentiate into subpopulations that specialize in different parts of a catabolic pathway.

We see our study of single-cell assimilation as a contribution towards understanding the extent and the relevance of metabolic differences between genetically identical cells. Using stable isotope-labeled substrates in combination with single-cell mass spectrometry and mathematical modeling offers a powerful method for such an analysis. This method complements the more established approach of using transcriptional or translational fluorescent reporters alone. Furthermore, the method does not require genetic manipulation, and is thus applicable to all the diverse types of microbes that can be cultured in the laboratory. We expect that such studies will reveal how widespread metabolic diversity is in clonal populations, and how important it is for the activities and metabolic potential of these populations.

## Materials and methods

### Bacterial strains

All experiments were performed with *Escherichia coli* K-12 MG1655 [36] and its derivatives (see S2 Table). We also used a natural isolate of *Escherichia coli*, an enteroaggregative pathogenic strain 55989 (CRBIP14.5) [38], obtained from CRBIP-Institute Pasteur, Paris, France. A 2473 base-pair long dual reporter system was made by total synthesis by DNA2.0 in Menlo Park, CA, USA (S1 Sequence). More details are provided in S1 File, ‘Supplementary Methods’.

### Chemostat cultivation

Frozen strains were streaked once on LB agar plates to obtain single colonies. A single colony was inoculated overnight at 37°C in minimal media containing 1x M9 salts (Sigma-Aldrich), 1 mM MgSO_4_ (Fluka) and 0.1 mM CaCl_2_ (Sigma-Aldrich), supplemented with 3 mM D-glucose (Glc) (Sigma), 3 mM L-arabinose (Ara) (Sigma-Aldrich) and 5% (v/v) LB complex broth (total 4 ml) (Sigma-Aldrich). The 1000-fold diluted overnight cultures were used to inoculate precultures (total 4 ml) in defined minimal media containing 47.76 mM Na_2_HPO_4_ (Sigma), 23.6 mM KH_2_PO_4_, 8.56 mM NaCl, 20.2 μM NH_4_Cl, 1 mM MgSO_4_ (all from Fluka) and 0.1 mM CaCl_2_. Sugar concentration was as follows: 10 μM Glc and 10 μM Ara in carbon-limited media, 3 mM Glc and 3 mM Ara in carbon-excess media, solely 20 μM Glc or solely 20 μM Ara for single-substrate studies. Carbon-excess chemostat cultures were nitrogen-limited (limitation in NH_4_Cl) thus all chemostats had similar bacterial population size. The precultures were grown for 12 hours until mid-exponential phase (for growth data see S5B Fig), and 1 ml of precultures was used to inoculate glass mini-chemostats [2]. The dilution rate for chemostats operation was increased in 2 steps, from the minimal speed of the inflow pump (IPC-N model, Ismatec, IDEX Health & Science, Germany) until 0.15 h^-1^ was reached after 14 h. Chemostats were harvested after 5 volume changes, which is the minimum number of volume changes suggested for reaching the steady-state in the respective mini-chemostat system [2, 46]. The outflow pump (IP model, Ismatec, IDEX Health & Science, Germany) was attached to a syringe that was positioned at the liquid surface in the chemostats, and operated at 20 ml per minute. This kept a constant volume and supplied the culture with flow of filter-sterilized, water-saturated air [2]. Each mini-chemostat contained a magnetic stir bar inside; a rack with the mini-chemostats was placed in a waterbath at 37°C, and the waterbath was placed on a magnetic stirrer. Continuous supply of air-bubbles together with magnetic stirring facilitated adequate mixing of the bacterial cultures in the mini-chemostat system.

The volume of chemostat cultures was in total 5.6 ml and the pump rate was set to 0.84 ml h^-1^, which means that bacterial generation time was 4.62 hours, and that chemostats needed to operate 6.67 hours to get one volume change. pH during the experiments remained constant at pH = 7.1. The average OD_600_ of cultures grown in carbon-limited and carbon-excess chemostats was 0.008 (with the standard errors of the mean of 0.001 and 0.0007, respectively). Colony count was on average 2.45 x 10^6^ ml^-1^ in carbon-limited and 2.4 x 10^6^ ml^-1^ in nitrogen-limited carbon-excess chemostats.

### Incubation with the stable isotope-labeled sugars in chemostats

After completing five volume changes in mini-chemostats, media-flow was switched to media bottles containing stable isotope-labeled carbon sources, which were in the same concentrations as in the unlabeled media. The defined minimal medium was supplemented with D-glucose labeled with deuterium ^2^H (D-[UL-^2^H_12_]-glucose, product number 616338, 97% labeling, Sigma-Aldrich, Switzerland) and L-arabinose labeled with ^13^C (L-[UL-^13^C_5_]-arabinose, 99% labeling, ANAWA Trading SA, Switzerland). The cultures were run for additional 3 hours (45% of volume change, corresponding to 65% of generation time) in the mini-chemostats and then harvested. As a control, we also prepared cultures of strain NN114 grown in carbon-limited chemostats without incubation with stable isotope-labeled sugars. More details are provided in S1 File, sections 'Fixation of bacterial samples', 'Maximal incorporation of the stable isotope-labeled sugars in chemostats', and 'AOC contamination in the chemostat setup'.

### Incubation with the stable isotope-labeled sugars in batch cultures

Batch cultures of strain NN114 were grown in media supplemented with 3 mM Glc and 1.5 mM Ara; other components of the medium were as used for chemostat cultivation. Exponential populations had starting A_600_ (absorbance at 600 nm) of 0.001 and total volume of 200 μl, and were incubated in the plate-reader Eon (BioTek) at 37°C, with continuous shaking. The populations were growing for 9 h until the cells assimilated approximately half of supplemented Glc leaving 1.5 mM unlabeled Glc and Ara in the cell suspension. Then we added isotopically labeled sugars ^2^H-Glc and ^13^C-Ara to a final concentration of 1.5 mM each, by adding 2 μl of a 150 mM stock solution to the cell suspension. This resulted in a medium composition of 3 mM Glc and 3 mM Ara, both labeled approximately with 50% ^2^H and ^13^C, respectively, at the time when the stable isotope incubation started. We used absorbance, glucose, and yield measurements to more precisely estimate the isotopic labeling of glucose in each replicate. After 36 minutes, the cultures were fixed in formaldehyde and stained as described in S1 File, section ‘Fixation of bacterial samples’, and applied on filters for NanoSIMS measurements, as described in S1 File, section ‘Sample preparation for NanoSIMS’. As a control, we also prepared reference filters with unlabeled cells grown in the same conditions, which we used to determine “background” or natural isotopic composition and “filter-reference” (see the section below, ‘NanoSIMS data analysis’). This filter contained cells of strain NN114 growing in batch culture with Glc and Ara, without incubation with stable isotope-labeled sugars.

### NanoSIMS data analysis

By using NanoSIMS, we collected count data for the mass fragments ^1^H, ^2^H, ^12^C and ^13^C, together with the detection of secondary electrons (Esi) that provides information about surface topography (Fig 4). Images were processed using Matlab-based script Look@NanoSIMS [66]. The images were first corrected for a possible drift of the sample during the measurement, and then the counts in each pixel were accumulated over the measured z-planes. We discarded planes that were acquired incorrectly. Cell outlines (region of interest, ROI) were drawn according to secondary electron images. The accumulated counts *c* were averaged over the area of a cell, and the uncorrected atom fractions for carbon, *X* (^13^C)_cell/uncorrected_, and hydrogen, *X* (^2^H)_cell/uncorrected_, were calculated as ratios:

$$X{{(}^{13}C)}_{cell/uncorrected}=c{[}^{13}C]/(c{[}^{13}C]+c{[}^{12}C])$$

$$X{{(}^{2}H)}_{cell/uncorrected}=c{[}^{2}H]/(c{[}^{2}H]+c{[}^{1}H])$$

To infer measurement error, Poisson percentage and standard errors of the ratio were determined by Look@NanoSIMS. These statistical errors represent the theoretical precision of the reported mean ratio, and the exact calculation is described in [66]. For statistical analysis of stable isotope-labeled cells we used only those cells with Poisson percentage errors below 10% for *c*[^2^H]/*c*[^1^H], and below 1% for *c*[^13^C]/*c*[^12^C] (S12 Fig).

For each image we calculated atom fractions outside ROI, *X* (^13^C)_filter/cells,_ *X* (^2^H)_filter/cells._ We also measured the reference filter with unlabeled cells to calculate atom fractions outside ROI (*X* (^13^C)_filter/reference_, *X* (^2^H)_filter/reference_) and atom fractions of the unlabeled cells as the background isotopic composition (*X* (^13^C)_cells/natural_ = 0.009928, *X* (^2^H)_cells/natural_ = 0.0002642).

For each image, the atom fractions were normalized to correct for day-to-day variation of the instrument by subtracting from the uncorrected atom fractions the difference between the measured atom fractions outside ROI of the analyzed filter (*X* (^13^C)_filter/cells_, *X* (^2^H)_filter/cells_), and atom fractions outside ROI of the reference filter (*X* (^13^C)_filter/reference_, *X* (^2^H)_filter/reference_) as:

$$X{{(}^{13}C)}_{cell}=X{{(}^{13}C)}_{cell/uncorrected}-[X{{(}^{13}C)}_{filter/cells}\;\text{-}\;X{{(}^{13}C)}_{filter/reference}]$$

$$X{{(}^{2}H)}_{cell}=X{{(}^{2}H)}_{cell/uncorrected}-[X{{(}^{2}H)}_{filter/cells}\;\text{-}\;X{{(}^{2}H)}_{filter/reference}]$$

The obtained corrected atom fractions (*X* (^13^C)_cell_, *X* (^2^H)_cell_) were used to calculate the excess atom fractions (*X*^*E*^ (^13^C)_cell_, *X*^*E*^ (^2^H)_cell_) by subtracting the background isotopic composition:

$$X^{E}{{(}^{13}C)}_{cell}=X{{(}^{13}C)}_{cell}\;\text{-}\;X{{(}^{13}C)}_{cells/natural}$$

$$X^{E}{{(}^{2}H)}_{cell}=X{{(}^{2}H)}_{cell}\;\text{-}\;X{{(}^{2}H)}_{cells/natural}$$

More details are provided in S1 File, sections 'Sample preparation for NanoSIMS', and 'NanoSIMS measurements'.

### Analysis of filters with fluorescence microscopy and NanoSIMS

#### Fluorescence microscopy and analysis

The fluorescence images of cells placed on NanoSIMS filters were recorded with the fluorescence lamp intensity set on 100% and exposure time of 1 s for GFP, RFP and DAPI filters (technical details in S1 File, ‘Fluorescence microscopy’), and with binning settings at 2x2. Phase contrast images could not be made due to the filter. We measured autofluorescence of strain 55989 on the NanoSIMS filters. For the image analysis, the cell outline was determined by DAPI, mCherry or GFP fluorescence for images taken from the NanoSIMS filters. A cell's fluorescence was first defined as a mean pixel value of the center of the cell corrected for the filter background, which was measured from an area outside cells on the same position. Finally, average GFP and mCherry fluorescence values from the cells of strain 55989 were subtracted as cell autofluorescence, and these final fluorescence values were analyzed further.

#### Overlap with NanoSIMS images

For the comparisons of fluorescence and NanoSIMS datasets (S1 Dataset), only those cells that displayed complete overlap between the two images were used (Fig 4A). To calculate ‘transcriptional specialization’ and ‘sugar specialization’ of individual cells (as plotted in Fig 4C) we did the following: we worked with the mCherry and GFP fluorescence after correcting for background and autofluorescence as described above, as well as excess atom fractions *X*^*E*^(^13^C)_cell_ and *X*^*E*^(^2^H)_cell_. Then, for each field of view (as shown in Fig 4A) we standardized these values to obtain mean = 0 and standard deviation = 1 for each of the four datasets. These standardized values were then combined into one dataset, and normalized to the range [0, 1]. Normalized values were used to calculate fluorescence specialization (‘transcriptional specialization’) as

$$\left(\mathrm{mCherry}\;{\mathrm{fluorescence}}_{\mathrm{norm}}\;\text{-}\;\mathrm{GFP}\;\mathrm{fluorescenc}e_{\mathrm{norm}}\right)/\left(\mathrm{mCherry}\;\mathrm{fluorescenc}e_{\mathrm{norm}}+\mathrm{GFP}\;\mathrm{fluorescenc}e_{\mathrm{norm}}\right)$$

and isotope specialization (‘sugar specialization’) as

$$\left(X^{E}{}_{norm}{{(}^{2}H)}_{cell}\;\text{-}\;X^{E}{}_{norm}{{(}^{13}C)}_{cell})/(X^{E}{}_{norm}{{(}^{2}H)}_{cell}+X^{E}{}_{norm}{{(}^{13}C)}_{cell}\right)$$

plotted in Fig 4C.

## Supporting information

S1 Fig Influence of the concentration of nitrogen source on the growth in carbon-deficient batch cultures. We tested whether altering the concentration of the nitrogen source NH_4_Cl significantly influences growth of the strain MG1655 in media containing micromolar concentrations of carbon sources. Stationary phase precultures were diluted 1 to 100 into 24-well plates, incubated at 37°C, and growth was measured by a plate reader (Synergy Mx, BioTek) as absorbance at 600 nm, A_600_. Records were taken every 15 minutes in total for 35 hours, and background was subtracted before further analysis of growth data. There were no significant growth differences when using media with different concentrations of NH_4_Cl. One can assume that the growth in media containing 10 μM Glc and 10 μM Ara is not limited by nitrogen, thus robust to changes in nitrogen source concentration. (Error bars present standard deviation between four replicates.)
(TIFF)

S2 Fig Bacterial growth on AOC (‘assimilable organic carbon’). Four replicate cultures of the MG1655 strain were grown in glass culture tubes containing M9 medium without any supplemented sugar. As determined by an increase in the CFU count per ml of the culture between ‘day 0’ and ‘day 1’, AOC can support growth of about 1.6 x 10^6^ cells/ml. Error bars present standard error of the mean from 4 biological replicates, with each replicate value averaged over 4 technical samples. The experiment is described in S1 File, section 'Bacterial growth on AOC'.
(TIFF)

S3 Fig Decreasing fraction of unlabeled sugars in chemostats after switch to media containing labeled sugars. Here we show the decreasing fraction of unlabeled glucose (blue curve) in nitrogen-limited, carbon-excess chemostats. From this curve we calculated the average fraction of unlabeled glucose that a cell experienced during the 3 hour-labeling period in chemostats (red line). This average fraction of unlabeled glucose is the integral of the blue curve during the 3 hour-labeling period, divided by the labeling period.
(TIFF)

S4 Fig Sugar assimilation in the pathogenic strain 55989. The pattern of assimilation of arabinose and glucose in the enteroaggregative *Escherichia coli* (EAEC) pathogenic strain 55989 was similar to the results obtained for the laboratory strain (Fig 1B). The assimilation of both isotopes in EAEC was significantly correlated and positive (Table 1). We did not observe that the level of metabolic specialization in EAEC was more pronounced than in the laboratory strain NN114. Statistical analysis revealed differences between the assimilation of ^13^C-arabinose and ^2^H-glucose in the clonal EAEC cells and the NN114 cells (Kolmogorov-Smirnov test: p-value = 0.046 for ^2^H excess atom fraction, and p-value = 0.001 for ^13^C excess atom fraction).
(TIFF)

S5 Fig Relevant growth characteristics of the EAEC strain 55989. The strains 55989 and MG1655 are phylogenetically closely related [38]. For example, the EAEC (enteroaggregative *E**scherichia* *c**oli*) strain 55989 has the PTS-glucose system as well as the AraE system encoded in its genome. By using NCBI BLAST [67] we identified that the promoter regions of the genes *ptsG*, *araE* and *rpsM* in the MG1655 strain (according to the sequences defined in the plasmid library [45]) are 100% identical with the corresponding EAEC sequences. Furthermore, the *ptsG* gene has 99% identity with the respective sequence in the EAEC strain, *araE* has 99% identity, and *rpsM* has 100% identity. Overnight grown cultures were diluted 1 to 100 into 24-well plates, and growth was recorded by a plate reader as A_600_ (the same setup as used in S1 Fig). **(A)** We used the plate-reader to show that the EAEC isolate can grow under laboratory conditions, in M9 minimal media with arabinose and/or glucose supplemented. (Error bars present standard deviation between 3 replicates for mixed-carbon, and 4 replicates for single carbon source conditions.) **(B)** We assessed whether growth characteristics of the EAEC strain are different than the NN114 strain (MG1655-derived strain) under the same nutrient concentrations as used in carbon-limited chemostats, in media containing 10 μM Glc and 10 μM Ara. We computed maximum growth rate μ_MAX_ on 10 μM Glc and 10 μM Ara for both strains. μ_MAX_ was defined as the maximal value of slopes calculated as ln-transformed average values over 3 time points, i.e. μ_MAX_ = 0.575 h^-1^ for strain NN114 measured between t_1_ = 5.25 h and t_2_ = 5.75 h; μ_MAX_ = 0.427 h^-1^ for the EAEC strain measured between t_1_ = 5 h and t_2_ = 5.5 h. (Error bars present standard deviation between 5 EAEC replicates and 4 replicates of strain NN114.)
(TIFF)

S6 Fig Estimated growth rates on glucose and arabinose in carbon-limited chemostats. Model values for growth rate on glucose, mean = 0.010 h^-1^, CV = 0.880; and on arabinose mean = 0.017 h^-1^, CV = 0.781. Model values for total estimated growth rate (Fig 2B), mean = 0.037 h^-1^, CV = 0.724.
(TIFF)

S7 Fig Cell-to-cell variation in growth rates in chemostats and batch cultures. We determined coefficients of variation (CVs) in growth rate in mixed-carbon environments. CVs are shown for growth on glucose (6 replicates; average CV = 0.858) and for total estimated growth (growth on glucose, arabinose and AOC) in carbon-limited chemostats (average CV = 0.723), and for growth on glucose in carbon-excess chemostats (nitrogen-limited, 2 replicates; average CV = 0.782) and carbon-excess batch cultures (3 replicates; average CV = 0.221). Error bars show standard error of the mean. Variation in growth rate was more than 3 times lower in the batch cultures than in the chemostats. For the analysis of isotope enrichments and calculations of growth rates in carbon-limited chemostats, carbon-excess chemostats, and carbon-excess batch cultures see S2 File, ‘Mathematical Model’.
(TIFF)

S8 Fig Expression of the transcriptional reporters for arabinose transporters and metabolic genes growing in batch cultures. Flow cytometry measurements of transcriptional reporters for arabinose transporters AraE and AraF, as well as an arabinose metabolic gene AraB and a regulator gene AraC are shown for two biological replicates (blue and green line) per each condition. In all graphs, the promoterless strain MG1655 pUA66 is depicted in grey, and GFP distributions of biological replicates of analyzed reporter-strains are depicted in different colors. The flow cytometer PAS-III Partec was used for analysis of the expression of *araE*, *araB* and *araC* in minimal media supplemented with 0.015 mM Ara, 0.15 mM Ara or 1.5 mM Ara; FACSCalibur was used in all other flow cytometry measurements. The expression of *araE* under intermediate concentrations of arabinose has previously been described to follow all-or-none response [68]. Under such conditions only a fraction of cells takes up arabinose and increased cytoplasmic levels of arabinose in these cells lead to the induction of genes involved in arabinose utilization [68, 69]. The expression of the *araE* reporter in bacterial populations growing solely on 0.015 mM Ara varied over three orders of magnitude. In addition to this cell-to-cell variation *within* replicate culture in the expression of the transcriptional reporter, we also observed marked variation in fluorescence *between* different replicate cultures (compare the green and blue histogram). This latter observation is consistent with the results shown in Fig 3B for 20 μM (i.e. 0.020 mM) arabinose, where we observed marked differences between replicate cultures in the expression of the *araE* and *ptsG* reporter. Moreover, the expression of the *araE* reporter was repressed when only glucose was present in the medium, and induced upon high concentration of arabinose in the medium. In contrast, the expression of the *araF* reporter was a less informative indicator of arabinose transport. Analysis of the *araB* reporter indicated expression patterns similar to the *araE* reporter, but the *araB* reporter showed a smaller dynamic range of the expression in bacterial populations growing with 0.15 mM or 1.5 mM Ara. The expression of ParaC-*gfp* was only slightly different between environments with various concentrations of arabinose as a sole carbon source. In summary, expression of the *araE* reporter varied accordingly to presence or absence of arabinose; hence we used this ParaE-*gfp* reporter for the main experiments.
(TIFF)

S9 Fig Expression of the reporters for glucose and arabinose uptake and metabolism in carbon-deficient conditions. We used plasmid-based promoter-reporters [45], and here plotted the results from two independent replicates (depicted in different colors) for measurements of metabolic fluorescent reporters (background fluorescence in grey). As a control for GFP fluorescence we measured expression of the transcriptional reporter for ribosomal protein S13, encoded by the *rpsM* gene. Data were acquired by the flow cytometer PAS-III, and gated as indicated in S1 File, ‘Supplementary Methods’. **(A)** The strains were grown in the same conditions as in the setup with chromosomally integrated reporters (i.e. analysis of the NN114 strain), with addition of 50 μg/ml of kanamycin. **(B)** We were also interested in dynamic range of the expression of transcriptional reporters measured in batch cultures. A single colony of the strain harboring respective reporter was inoculated in minimal M9 medium containing 30 μM Glc and 30 μM Ara at 37°C, and 50 μg/ml of kanamycin. Stationary phase overnight precultures were diluted 1 to 10, incubated until they reached mid-exponential phase (5 hours) and measured with the flow cytometry. Under glucose and arabinose limitation in chemostats, the expression of the *ptsG* reporter was more variable, and the expression of the *mglB* reporter was up-regulated in comparison to the batch conditions. One can notice that besides the *ptsG* reporter, reporters for other genes encoding for glucose transporters were expressed in some cells (*malX*) or in the majority of cells (*mglB*) in carbon-limited, mixed-substrate chemostats. This means that the single-cell profile of sugar assimilation could depend as well on the expression of other sugar transporters that were not analyzed in details in this study.
(TIFF)

S10 Fig Dynamic range of the expression of the *ptsG* and *araE* reporters tested in different chemostat and batch conditions. We analyzed the expression of reporters for the arabinose transporter AraE and the glucose transporter PtsG by using a plasmid-based promoter-*gfp* reporter system [45]. Fluorescence measurements of the negative control–the promoterless strain MG1655 pUA66 –are depicted in grey; GFP distributions of biological replicates of analyzed reporter-strains are depicted in different colors. Data were filtered by using the autogating tool in SSC vs. FSC pseudo-color plots in FlowJo, and gated on 10,000–12,000 events. **(A)** Expression of the reporter system ParaE-*gfp* is depicted in green, 3 (2) replicates at 0.15 h^-1^ (0.35 h^-1^), and PptsG-*gfp* is depicted in red, 2 (2) replicates at 0.15 h^-1^ (0.35 h^-1^). The strains harboring a reporter system were grown in carbon-limited minimal media supplemented with 30 μM Glc and 30 μM Ara, for 5 volume changes at given dilution rates. Data were acquired with FACSCalibur for experiments done at 0.35 h^-1^, and with PAS-III for experiments done at 0.15 h^-1^. The expression of the transporters was different in the chemostats operated at 0.35 h^-1^ in comparison to the chemostats operated at 0.15 h^-1^ since gene expression pattern depends on the growth rate, i.e. on the dilution rate. **(B)** Expression of PptsG-*gfp* (red) and ParaE-*gfp* (green) was measured in early, middle and late exponential phase, in M9 minimal medium supplemented with 30 μM Glc and 30 μM Ara. The measurements of 2 replicates per condition were done with PAS-III. Variation in the expression of PptsG-*gfp* was the lowest in mid-exponential phase during growth on micromolar concentrations of sugars. The mean expression of ParaE-*gfp* increased in the course of batch growth. Two distinct subpopulations with different reporter expression profiles emerged in the late exponential phase, and the fractions of cells did not express the reporters above the background level. Additionally, the fluorescence was measured in the early exponential phase, in M9 minimal medium supplemented with 6 mM Glc and 6 mM Ara and measured with FACSCalibur, to show effect of carbon catabolite repression acting to down-regulate expression of the *araE* reporter. The measurements of PptsG-*gfp* and ParaE-*gfp* from the mid-exponential phase are the same data as in S9 Fig, but gated with a different method.
(TIFF)

S11 Fig Influence of the insertion site and the choice of a fluorescent gene on the reporters’ expression. We measured fluorescence of 3 replicates of strain NN114 (PptsG-*mCherry* and ParaE-*gfp* inserted in *att*HK022; same data as in Fig 3), 2 replicates of NN111 (PptsG-*gfp* and ParaE-*mCherry* inserted in *att*HK022), and 2 replicates of NN111-81 (PptsG-*gfp* and ParaE-*mCherry* inserted in *att*P21) grown in carbon-limited chemostats with 10 μM Glc and 10 μM Ara. We standardized fluorescence values for each strain because measurements were performed with different reporter systems. Our analysis provided no evidence that the expression of the reporters for sugar transporters depends on the fluorescent genes used or on the chromosomal insertion site for the reporter system, i.e. a Kruskal-Wallis test showed that distributions of the expression are not significantly different for the three strains (p-value = 0.497 for the *araE* reporter fluorescence and p-value = 0.074 for the *ptsG* reporter fluorescence).
(TIFF)

S12 Fig Inferring variability in NanoSIMS measurements. Each data point indicates the excess atom fractions *X*^*E*^ (^2^H)_cell_ (^2^H assimilation from labeled glucose) and *X*^*E*^ (^13^C)_cell_ (^13^C assimilation from labeled arabinose) of single cells in carbon-limited chemostats (data from Fig 1B), carbon-excess nitrogen-limited chemostats (data from Fig 1B) and carbon-excess batch cultures (data from S7 Fig). These measurements have Poisson percentage errors below 10% for *c*[^2^H]/*c*[^1^H] and below 1% for *c*[^13^C]/*c*[^12^C], and the indicated x-axis and y-axis error bars correspond to the Poisson standard errors for each cell measurement, determined by Look@NanoSIMS [66].
(TIFF)

S1 Table Maximal isotopic labeling in carbon-limited chemostats. (PDF)

S2 Table *Escherichia coli* strains and plasmids used in this study. (PDF)

S1 Sequence Sequence of the dual reporter system. (PDF)

S1 File Supplementary methods. (PDF)

S2 File Mathematical model. (PDF)

S1 Dataset Source data for figures and tables. (ZIP)
