# Supplementary figures and images for: Cell-to-cell variation and specialization in sugar metabolism in clonal bacterial populations
Source: PLoS Genet. 2017 Dec 18;13(12):e1007122. doi: 10.1371/journal.pgen.1007122 (PMC5773225; doi:10.1371/journal.pgen.1007122)

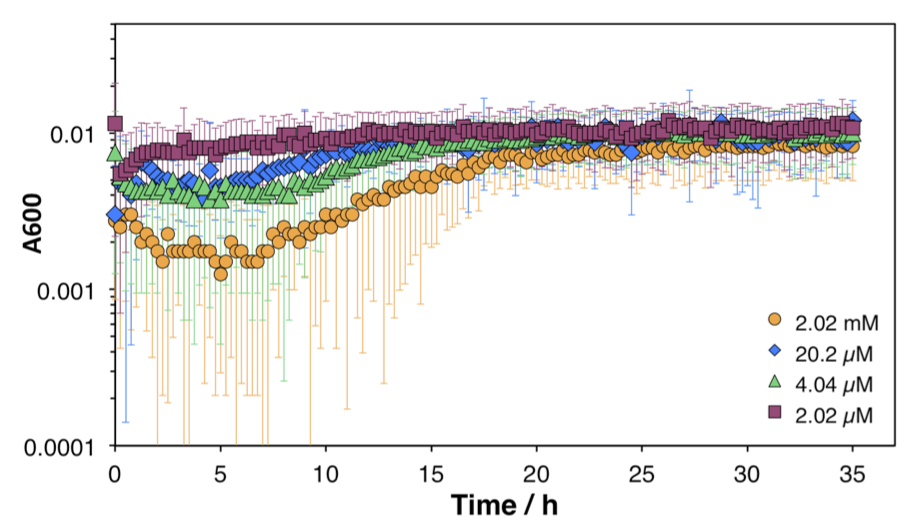

Supplement: S1 Fig — We tested whether altering the concentration of the nitrogen source NH4Cl significantly influences growth of the strain MG1655 in media containing micromolar concentrations of carbon sources. Stationary phase precultures were diluted 1 to 100 into 24-well plates, incubated at 37°C, and growth was measured by a plate reader (Synergy Mx, BioTek) as absorbance at 600 nm, A600. Records were taken every 15 minutes in total for 35 hours, and background was subtracted before further analysis of growth data. There were no significant growth differences when using media with different concentrations of NH4Cl. One can assume that the growth in media containing 10 μM Glc and 10 μM Ara is not limited by nitrogen, thus robust to changes in nitrogen source concentration. (Error bars present standard deviation between four replicates.) (TIFF) [file pgen.1007122.s001.tiff]

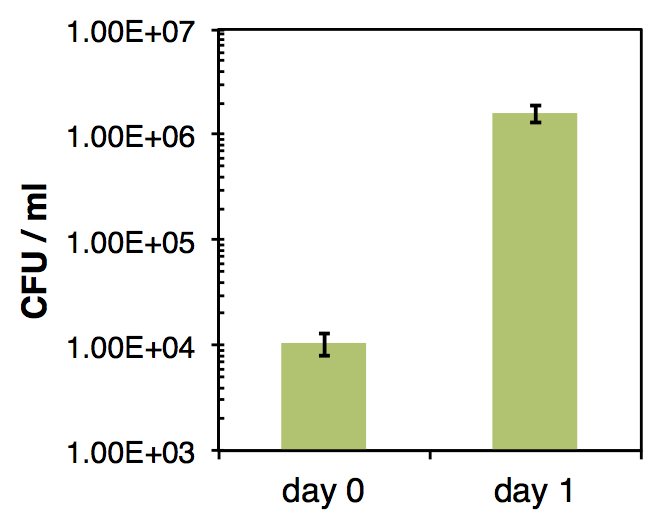

Supplement: S2 Fig — Four replicate cultures of the MG1655 strain were grown in glass culture tubes containing M9 medium without any supplemented sugar. As determined by an increase in the CFU count per ml of the culture between ‘day 0’ and ‘day 1’, AOC can support growth of about 1.6 x 10^6^ cells/ml. Error bars present standard error of the mean from 4 biological replicates, with each replicate value averaged over 4 technical samples. The experiment is described in S1 File, section 'Bacterial growth on AOC'. (TIFF) [file pgen.1007122.s002.tiff]

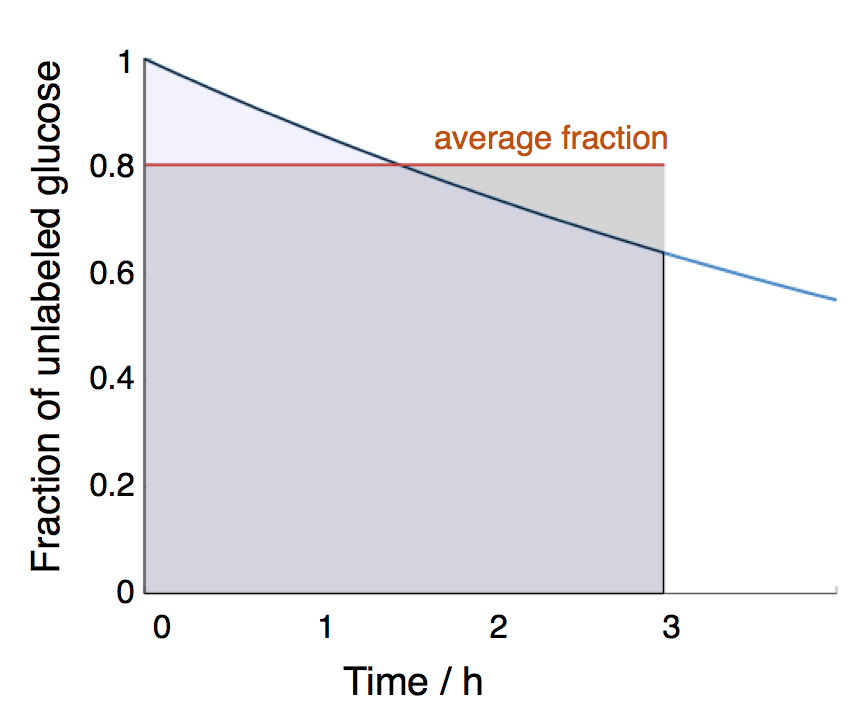

Supplement: S3 Fig — Here we show the decreasing fraction of unlabeled glucose (blue curve) in nitrogen-limited, carbon-excess chemostats. From this curve we calculated the average fraction of unlabeled glucose that a cell experienced during the 3 hour-labeling period in chemostats (red line). This average fraction of unlabeled glucose is the integral of the blue curve during the 3 hour-labeling period, divided by the labeling period. (TIFF) [file pgen.1007122.s003.tiff]

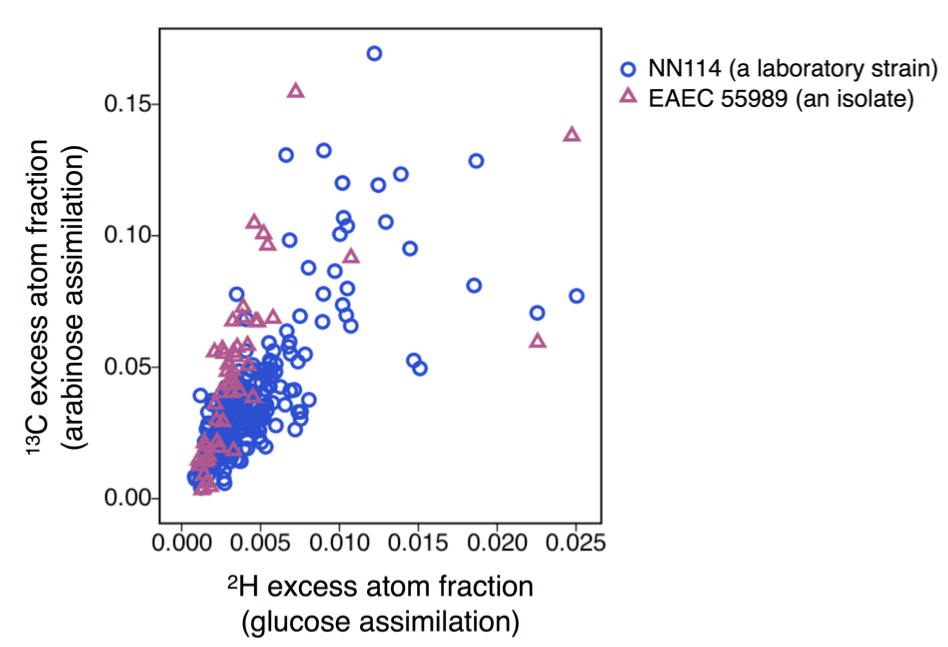

Supplement: S4 Fig — The pattern of assimilation of arabinose and glucose in the enteroaggregative Escherichia coli (EAEC) pathogenic strain 55989 was similar to the results obtained for the laboratory strain (Fig 1B). The assimilation of both isotopes in EAEC was significantly correlated and positive (Table 1). We did not observe that the level of metabolic specialization in EAEC was more pronounced than in the laboratory strain NN114. Statistical analysis revealed differences between the assimilation of 13C-arabinose and 2H-glucose in the clonal EAEC cells and the NN114 cells (Kolmogorov-Smirnov test: p-value = 0.046 for 2H excess atom fraction, and p-value = 0.001 for 13C excess atom fraction). (TIFF) [file pgen.1007122.s004.tiff]

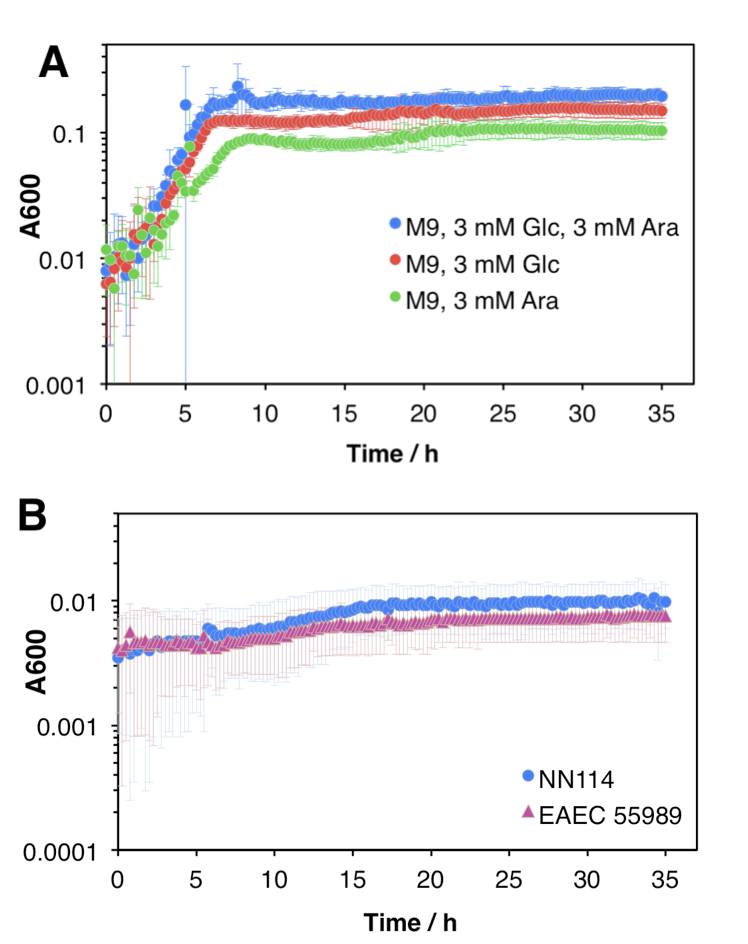

Supplement: S5 Fig — The strains 55989 and MG1655 are phylogenetically closely related [38]. For example, the EAEC (enteroaggregative Escherichia coli) strain 55989 has the PTS-glucose system as well as the AraE system encoded in its genome. By using NCBI BLAST [67] we identified that the promoter regions of the genes ptsG, araE and rpsM in the MG1655 strain (according to the sequences defined in the plasmid library [45]) are 100% identical with the corresponding EAEC sequences. Furthermore, the ptsG gene has 99% identity with the respective sequence in the EAEC strain, araE has 99% identity, and rpsM has 100% identity. Overnight grown cultures were diluted 1 to 100 into 24-well plates, and growth was recorded by a plate reader as A600 (the same setup as used in S1 Fig). (A) We used the plate-reader to show that the EAEC isolate can grow under laboratory conditions, in M9 minimal media with arabinose and/or glucose supplemented. (Error bars present standard deviation between 3 replicates for mixed-carbon, and 4 replicates for single carbon source conditions.) (B) We assessed whether growth characteristics of the EAEC strain are different than the NN114 strain (MG1655-derived strain) under the same nutrient concentrations as used in carbon-limited chemostats, in media containing 10 μM Glc and 10 μM Ara. We computed maximum growth rate μMAX on 10 μM Glc and 10 μM Ara for both strains. μMAX was defined as the maximal value of slopes calculated as ln-transformed average values over 3 time points, i.e. μMAX = 0.575 h-1 for strain NN114 measured between t1 = 5.25 h and t2 = 5.75 h; μMAX = 0.427 h-1 for the EAEC strain measured between t1 = 5 h and t2 = 5.5 h. (Error bars present standard deviation between 5 EAEC replicates and 4 replicates of strain NN114.) (TIFF) [file pgen.1007122.s005.tiff]

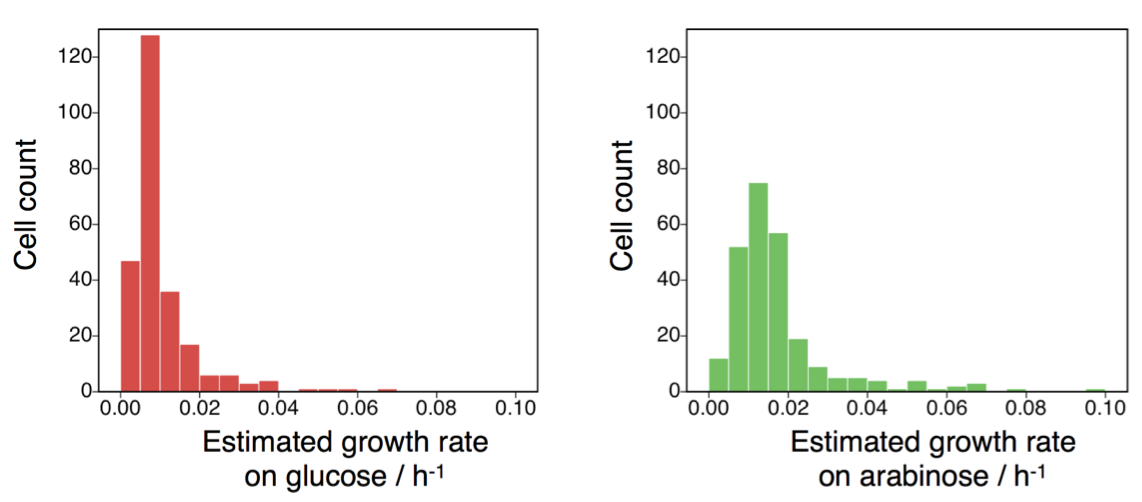

Supplement: S6 Fig — Model values for growth rate on glucose, mean = 0.010 h-1, CV = 0.880; and on arabinose mean = 0.017 h-1, CV = 0.781. Model values for total estimated growth rate (Fig 2B), mean = 0.037 h-1, CV = 0.724. (TIFF) [file pgen.1007122.s006.tiff]

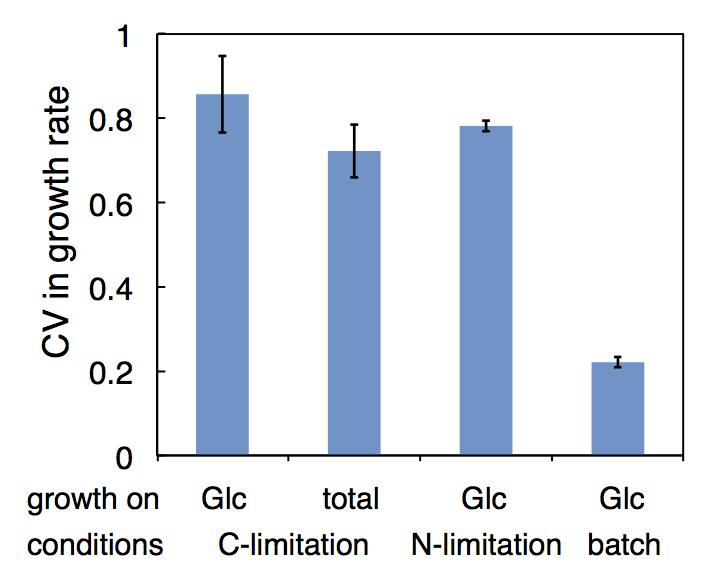

Supplement: S7 Fig — We determined coefficients of variation (CVs) in growth rate in mixed-carbon environments. CVs are shown for growth on glucose (6 replicates; average CV = 0.858) and for total estimated growth (growth on glucose, arabinose and AOC) in carbon-limited chemostats (average CV = 0.723), and for growth on glucose in carbon-excess chemostats (nitrogen-limited, 2 replicates; average CV = 0.782) and carbon-excess batch cultures (3 replicates; average CV = 0.221). Error bars show standard error of the mean. Variation in growth rate was more than 3 times lower in the batch cultures than in the chemostats. For the analysis of isotope enrichments and calculations of growth rates in carbon-limited chemostats, carbon-excess chemostats, and carbon-excess batch cultures see S2 File, ‘Mathematical Model’. (TIFF) [file pgen.1007122.s007.tiff]

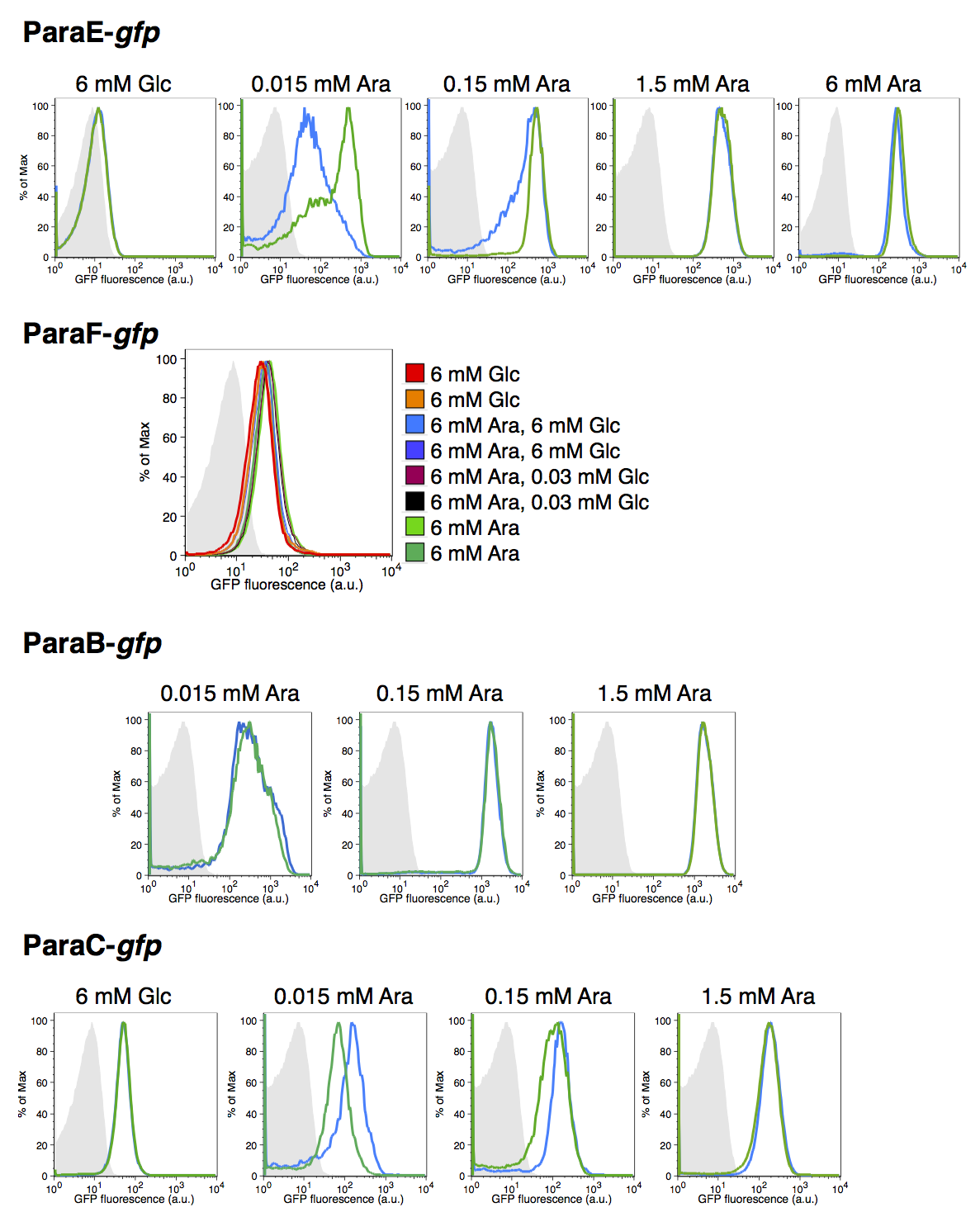

Supplement: S8 Fig — Flow cytometry measurements of transcriptional reporters for arabinose transporters AraE and AraF, as well as an arabinose metabolic gene AraB and a regulator gene AraC are shown for two biological replicates (blue and green line) per each condition. In all graphs, the promoterless strain MG1655 pUA66 is depicted in grey, and GFP distributions of biological replicates of analyzed reporter-strains are depicted in different colors. The flow cytometer PAS-III Partec was used for analysis of the expression of araE, araB and araC in minimal media supplemented with 0.015 mM Ara, 0.15 mM Ara or 1.5 mM Ara; FACSCalibur was used in all other flow cytometry measurements. The expression of araE under intermediate concentrations of arabinose has previously been described to follow all-or-none response [68]. Under such conditions only a fraction of cells takes up arabinose and increased cytoplasmic levels of arabinose in these cells lead to the induction of genes involved in arabinose utilization [68, 69]. The expression of the araE reporter in bacterial populations growing solely on 0.015 mM Ara varied over three orders of magnitude. In addition to this cell-to-cell variation within replicate culture in the expression of the transcriptional reporter, we also observed marked variation in fluorescence between different replicate cultures (compare the green and blue histogram). This latter observation is consistent with the results shown in Fig 3B for 20 μM (i.e. 0.020 mM) arabinose, where we observed marked differences between replicate cultures in the expression of the araE and ptsG reporter. Moreover, the expression of the araE reporter was repressed when only glucose was present in the medium, and induced upon high concentration of arabinose in the medium. In contrast, the expression of the araF reporter was a less informative indicator of arabinose transport. Analysis of the araB reporter indicated expression patterns similar to the araE reporter, but the araB reporter showed [file pgen.1007122.s008.tiff]

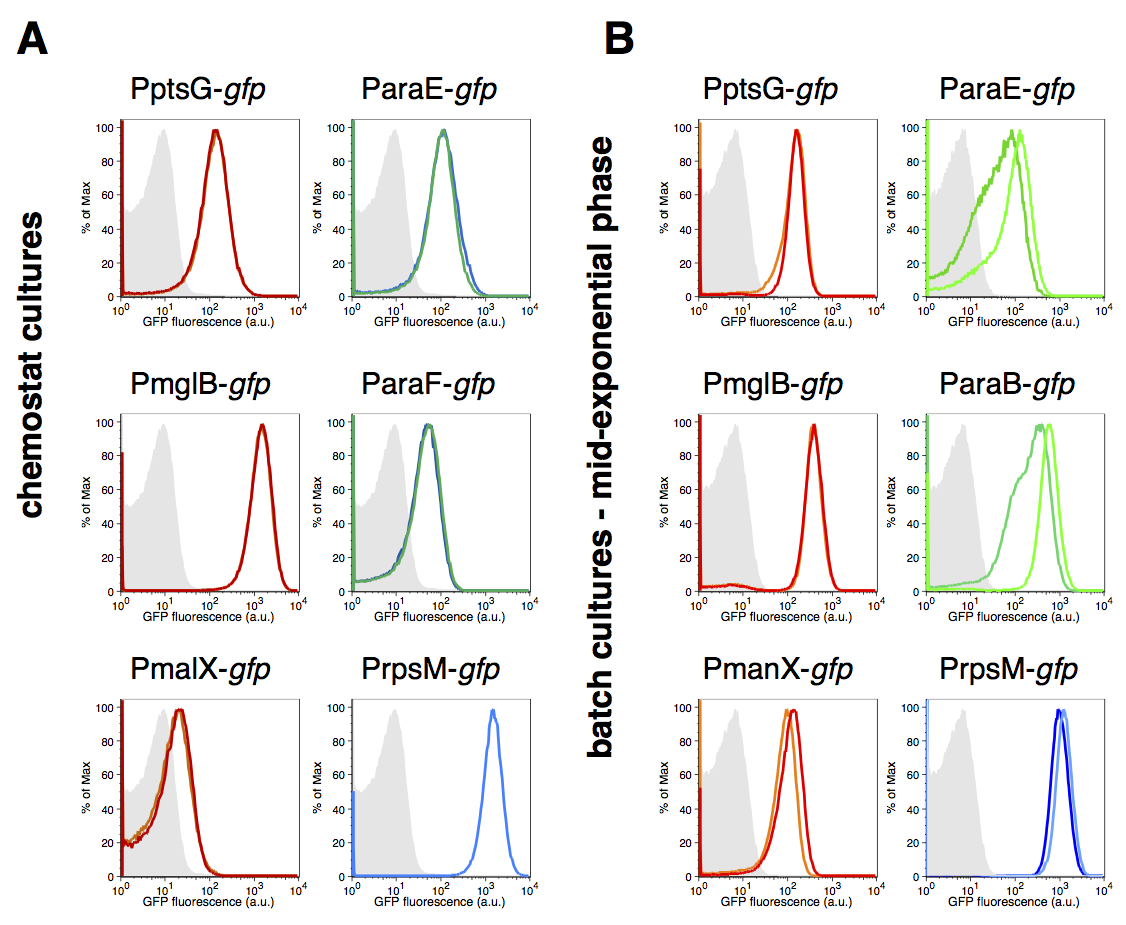

Supplement: S9 Fig — We used plasmid-based promoter-reporters [45], and here plotted the results from two independent replicates (depicted in different colors) for measurements of metabolic fluorescent reporters (background fluorescence in grey). As a control for GFP fluorescence we measured expression of the transcriptional reporter for ribosomal protein S13, encoded by the rpsM gene. Data were acquired by the flow cytometer PAS-III, and gated as indicated in S1 File, ‘Supplementary Methods’. (A) The strains were grown in the same conditions as in the setup with chromosomally integrated reporters (i.e. analysis of the NN114 strain), with addition of 50 μg/ml of kanamycin. (B) We were also interested in dynamic range of the expression of transcriptional reporters measured in batch cultures. A single colony of the strain harboring respective reporter was inoculated in minimal M9 medium containing 30 μM Glc and 30 μM Ara at 37°C, and 50 μg/ml of kanamycin. Stationary phase overnight precultures were diluted 1 to 10, incubated until they reached mid-exponential phase (5 hours) and measured with the flow cytometry. Under glucose and arabinose limitation in chemostats, the expression of the ptsG reporter was more variable, and the expression of the mglB reporter was up-regulated in comparison to the batch conditions. One can notice that besides the ptsG reporter, reporters for other genes encoding for glucose transporters were expressed in some cells (malX) or in the majority of cells (mglB) in carbon-limited, mixed-substrate chemostats. This means that the single-cell profile of sugar assimilation could depend as well on the expression of other sugar transporters that were not analyzed in details in this study. (TIFF) [file pgen.1007122.s009.tiff]

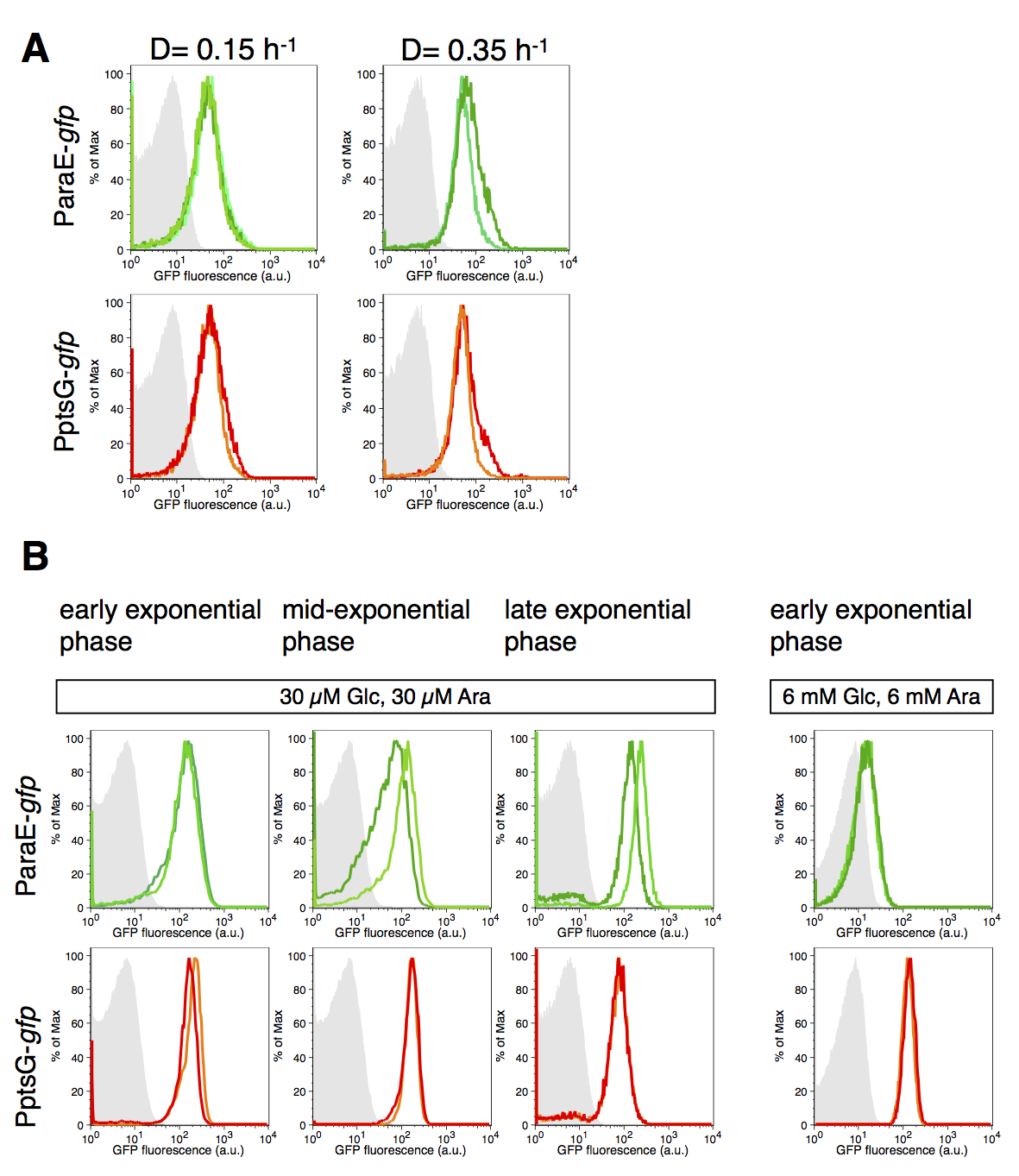

Supplement: S10 Fig — We analyzed the expression of reporters for the arabinose transporter AraE and the glucose transporter PtsG by using a plasmid-based promoter-gfp reporter system [45]. Fluorescence measurements of the negative control–the promoterless strain MG1655 pUA66 –are depicted in grey; GFP distributions of biological replicates of analyzed reporter-strains are depicted in different colors. Data were filtered by using the autogating tool in SSC vs. FSC pseudo-color plots in FlowJo, and gated on 10,000–12,000 events. (A) Expression of the reporter system ParaE-gfp is depicted in green, 3 (2) replicates at 0.15 h-1 (0.35 h-1), and PptsG-gfp is depicted in red, 2 (2) replicates at 0.15 h-1 (0.35 h-1). The strains harboring a reporter system were grown in carbon-limited minimal media supplemented with 30 μM Glc and 30 μM Ara, for 5 volume changes at given dilution rates. Data were acquired with FACSCalibur for experiments done at 0.35 h-1, and with PAS-III for experiments done at 0.15 h-1. The expression of the transporters was different in the chemostats operated at 0.35 h-1 in comparison to the chemostats operated at 0.15 h-1 since gene expression pattern depends on the growth rate, i.e. on the dilution rate. (B) Expression of PptsG-gfp (red) and ParaE-gfp (green) was measured in early, middle and late exponential phase, in M9 minimal medium supplemented with 30 μM Glc and 30 μM Ara. The measurements of 2 replicates per condition were done with PAS-III. Variation in the expression of PptsG-gfp was the lowest in mid-exponential phase during growth on micromolar concentrations of sugars. The mean expression of ParaE-gfp increased in the course of batch growth. Two distinct subpopulations with different reporter expression profiles emerged in the late exponential phase, and the fractions of cells did not express the reporters above the background level. Additionally, the fluorescence was measured in the early exponential phase, in M9 minimal medium supplemented with 6 mM Glc and 6 [file pgen.1007122.s010.tiff]

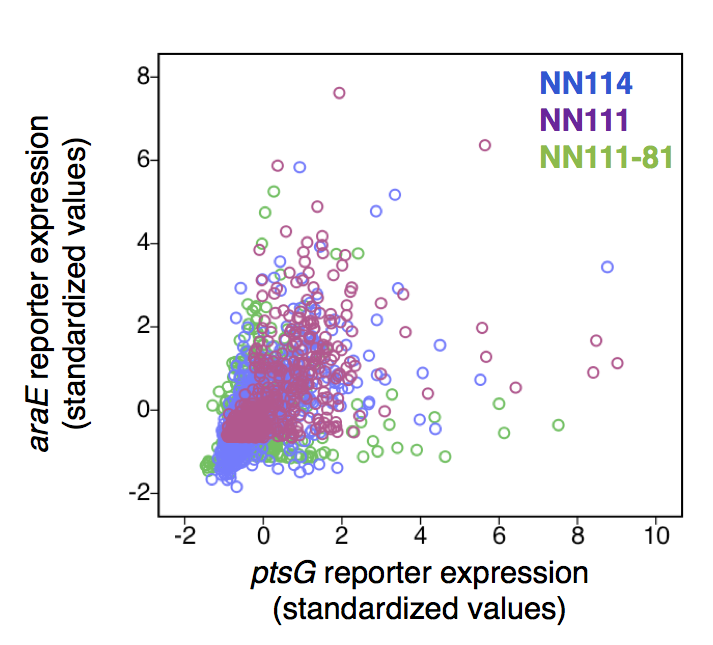

Supplement: S11 Fig — We measured fluorescence of 3 replicates of strain NN114 (PptsG-mCherry and ParaE-gfp inserted in attHK022; same data as in Fig 3), 2 replicates of NN111 (PptsG-gfp and ParaE-mCherry inserted in attHK022), and 2 replicates of NN111-81 (PptsG-gfp and ParaE-mCherry inserted in attP21) grown in carbon-limited chemostats with 10 μM Glc and 10 μM Ara. We standardized fluorescence values for each strain because measurements were performed with different reporter systems. Our analysis provided no evidence that the expression of the reporters for sugar transporters depends on the fluorescent genes used or on the chromosomal insertion site for the reporter system, i.e. a Kruskal-Wallis test showed that distributions of the expression are not significantly different for the three strains (p-value = 0.497 for the araE reporter fluorescence and p-value = 0.074 for the ptsG reporter fluorescence). (TIFF) [file pgen.1007122.s011.tiff]

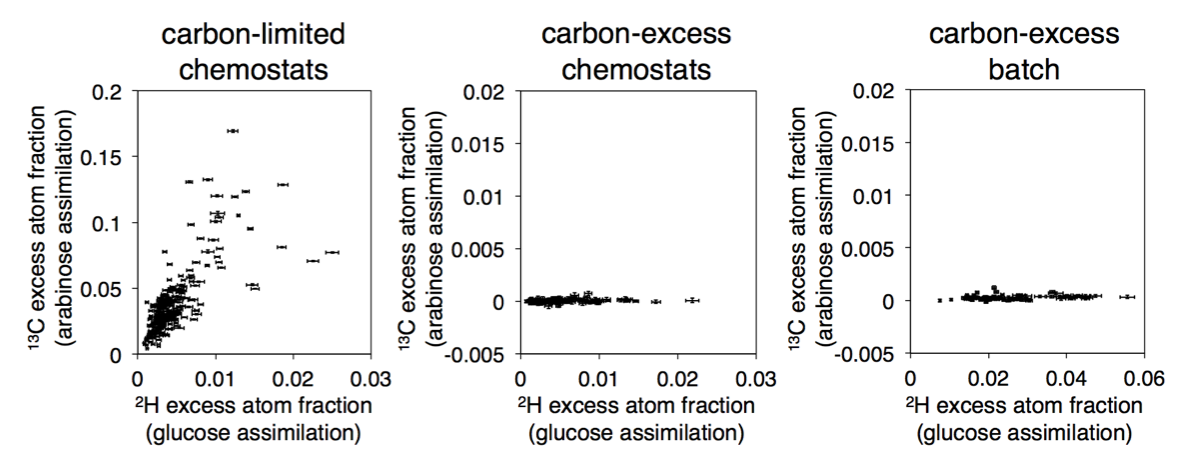

Supplement: S12 Fig — Each data point indicates the excess atom fractions XE (2H)cell (2H assimilation from labeled glucose) and XE (13C)cell (13C assimilation from labeled arabinose) of single cells in carbon-limited chemostats (data from Fig 1B), carbon-excess nitrogen-limited chemostats (data from Fig 1B) and carbon-excess batch cultures (data from S7 Fig). These measurements have Poisson percentage errors below 10% for c[2H]/c[1H] and below 1% for c[13C]/c[12C], and the indicated x-axis and y-axis error bars correspond to the Poisson standard errors for each cell measurement, determined by Look@NanoSIMS [66]. (TIFF) [file pgen.1007122.s012.tiff]
