# Supplementary material for: Cell-to-cell variation and specialization in sugar metabolism in clonal bacterial populations
Source: PLoS Genet. 2017 Dec 18;13(12):e1007122. doi: 10.1371/journal.pgen.1007122 (PMC5773225; doi:10.1371/journal.pgen.1007122)
Supplement: S1 Table — (PDF) [file pgen.1007122.s013.pdf]

Table S1. Maximal isotopic labeling in carbon-limited chemostats, measured after 6.9 volume turnovers, corresponding to 9.96 generations.

	*Mean level of maximal labeling			
Label	X^{E} (^{13}C)$_{cell}$	X^{E} (^{2}H)$_{cell}$	**CV in maximal labeling	N cells
20 μM ^{13}C-Ara	0.7074 ± 0.0101	/	0.082	33
20 μM ^{2}H-Glc	/	0.1386 ± 0.0047	0.190	32
10 μM ^{13}C-Ara and 10 μM ^{13}C-Glc	0.6874 ± 0.0056	/	0.087	113

*mean value ± standard error of the mean

**CV in ^{13}C (^{2}H) excess atom fractions is 8 times (4 times) lower for maximal isotopic labeling than for the measured populations growing in carbon-limited chemostats, which are presented in Fig 1B.
