# Supplementary material for: Cell-to-cell variation and specialization in sugar metabolism in clonal bacterial populations
Source: PLoS Genet. 2017 Dec 18;13(12):e1007122. doi: 10.1371/journal.pgen.1007122 (PMC5773225; doi:10.1371/journal.pgen.1007122)
Supplement: S2 Table — (PDF) [file pgen.1007122.s014.pdf]

Table S2. *Escherichia coli* strains and plasmids used in this study.

Strain or plasmid name	Characteristics	Source
MG1655	Wild-type *E.coli* K-12<br>F-, λ-, *ilvG*-, *rfb*-50, *rph*-1	Ackermann strain collection,<br>[Blattner et al., 1997]
55989	Enteroaggregative *E.coli* (EAEC)	CRBIP-Institut Pasteur, Paris, France,<br>[Touchon et al., 2009]
DH5α	Strain for plasmid propagation<br>F-, *glnV44*(AS), λ-, *deoR481*, *rfbC1*?, *gyrA96*(NalR), *recA1*, *endA1*, *thiE1*, *hsdR17*	Ackermann strain collection
BW25142	Strain for CRIM plasmid propagation<br>F-, *Δ(araD-araB)567*, *ΔlacZ4787*(::rrnB-3), *Δ(phoB-phoR)580*, λ^-, *galU95*, *ΔuidA4::pir-116*, *recA1*, *endA9*(del-ins)*::FRT*, *rph-1*, *Δ(rhaD-rhaB)568*, *hsdR514*	The Coli Genetic Stock Center,<br>Yale University,<br>[Haldimann and Wanner, 2001]
NN114	*att*HK022::PptsG-*mCherry* ParaE-*gfp::FRT*	This study
NN112	*att*HK022::ParaE-*mCherry* PrpsM-*gfp::FRT*	This study
NN113	*att*HK022::PptsG-*mCherry* PrpsM-*gfp::FRT*	This study
NN111	*att*HK022::ParaE-*mCherry* PptsG-*gfp::FRT*	This study
NN111-81	*att*P21::ParaE-*mCherry* PptsG-*gfp::FRT*	This study
MG1655 pGFP	pGDR11-Ptac-*gfp* in MG1655, AmpR	[Refardt et al., 2013]
MG1655 pRFP	pGDR11-Ptac-*mCherry* in MG1655, AmpR	Ackermann strain collection
MG1655 PptsG-*gfp*	Plasmid-based PptsG reporter in MG1655, KanR	Plasmid library [Zaslaver et al., 2006]
MG1655 ParaE-*gfp*	Plasmid-based ParaE reporter in MG1655, KanR	Plasmid library [Zaslaver et al., 2006]
MG1655 PrpsM-*gfp*	Plasmid-based PrpsM reporter in MG1655, KanR	Plasmid library [Zaslaver et al., 2006]
MG1655 PmglB-*gfp*	Plasmid-based PmglB reporter in MG1655, KanR	Plasmid library [Zaslaver et al., 2006]
MG1655 PmanX-*gfp*	Plasmid-based PmanX reporter in MG1655, KanR	Plasmid library [Zaslaver et al., 2006]
MG1655 ParaF-*gfp*	Plasmid-based ParaF reporter in MG1655, KanR	Plasmid library [Zaslaver et al., 2006]
MG1655 pUA66	Promoterless plasmid in MG1655, KanR	Plasmid library [Zaslaver et al., 2006]
pCP20	yeast Flp recombinase activity; *flp*, *bla*, *cat*, rep101ts	[Cherepanov and Wackernagel, 1995]
pNN111	ParaE-*mCherry*, PptsG-*gfp*, KanR, GentR, pUC ori	This study (the reporter construct made by DNA2.0, Menlo Park, CA, USA)
pNN112	ParaE-*mCherry*, PrpsM-*gfp*, KanR, GentR, pUC ori	This study
pNN113	PptsG-*mCherry*, PrpsM-*gfp*, KanR, GentR, pUC ori	This study
pNN114	PptsG-*mCherry*, ParaE-*gfp*, KanR, GentR, pUC ori	This study
pAH68-frt-chlor	pAH68 Δ*bla*::frt-*cat*-frt	Made by Tobias Bergmiller,<br>Guet strain collection
pAH81-frt-chlor	pAH81 Δ*bla*::frt-*cat*-frt	Made by Tobias Bergmiller,<br>Guet strain collection
pAH69	helper plasmid for integration in *att*HK022	[Haldimann and Wanner, 2001]
pAH121	helper plasmid for integration in *att*P21	[Haldimann and Wanner, 2001]

References

Blattner FR, Plunkett G, Bloch CA, Perna NT, Burland V, *et al.* (1997**)** The complete genome sequence of *Escherichia coli* K-12. *Science* **277:** 1453-1462

Cherepanov PP, Wackernagel W (1995) Gene disruption in *Escherichia coli*: TcR and KmR cassettes with the option of Flp-catalyzed excision of the antibiotic-resistance determinant. *Gene* **158**: 9–14

Haldimann A, Wanner BL (2001) Conditional-replication, integration, excision, and retrieval plasmid-host systems for gene structure-function studies of bacteria. *J Bacteriol* **183**: 6384–6393

Refardt D, Bergmiller T, Kümmerli R (2013) Altruism can evolve when relatedness is low: Evidence from bacteria committing suicide upon phage infection. *P Roy Soc B-Biol Sci* **280**: 20123035

Touchon M, Hoede C, Tenaillon O, Barbe V, Baeriswyl S, *et al.* (2009) Organised genome dynamics in the *Escherichia coli* species results in highly diverse adaptive paths. *PLOS Genet* **5**: e1000344

Zaslaver A, Kaplan S, Bren A, Jinich A, Mayo A, *et al.* (2009) Invariant distribution of promoter activities in *Escherichia coli*. *PLOS Comput Biol* **5**: e1000545
