# Supplementary material for: Cell-to-cell variation and specialization in sugar metabolism in clonal bacterial populations
Source: PLoS Genet. 2017 Dec 18;13(12):e1007122. doi: 10.1371/journal.pgen.1007122 (PMC5773225; doi:10.1371/journal.pgen.1007122)
Supplement: S1 Sequence — (PDF) [file pgen.1007122.s015.pdf]

Supplementary Sequence Information

Sequence S1.

>Sequence of the dual reporter system integrated in the strain NN111
TTATTTGTACAATTCATCCATACCATGGGTAATCCCAGCAGCTGTTACAAACTCAAGAAGGACCATGTG
GTCTCTCTTTTCGTTGGGATCTTTCGAAAGGGCAGATTGTGTGGACAGGTAATGGTTGTCTGGTAAAAG
GACAGGGCCATCGCCAATTGGAGTATTTTGTTGATAATGGTCTGCTAGTTGAACGCTTCCATCTTCAAT
GTTGTGTCTAATTTTGAAGTTAACTTTGATTCCATTCTTTTGTTTGTCTGCCATGATGTATACATTGTG
TGAGTTATAGTTGTATTCCAATTTGTGTCCAAGAATGTTTCCATCTTCTTTAAAATCAATACCTTTTAA
CTCGATTCTATTAACAAGGGTATCACCTTCAAACTTGACTTCAGCACGTGTCTTGTAGTTCCCGTCATC
TTTGAAAAATATAGTTCTTTCCTGTACATAACCTTCGGGCATGGCACTCTTGAAAAAGTCATGCTGTTT
CATATGATCTGGGTATCTCGCAAAGCATTGAAGACCATACGCGAAAGTAGTGACAAGTGTTGGCCATGG
AACAGGTAGTTTTCCAGTAGTGCAAATAAATTTAAGGGTAAGTTTTCCGTATGTTGCATCACCTTCACC
CTCTCCACTGACAGAAAATTTGTGCCCATTAACATCACCATCTAATTCAACAAGAATTGGGACAACTCC
AGTGAAAAGTTCTTCTCCTTTACTCATATGTATATCTCCTTCTTAAAGGATCC**TACGGATACCGGCAGC**
ATCAGCGATTTACCGACCTTTTGCAGGTTAGCAAATGCATTCTTAAACATAATTGAGAGTGCTCCTGAG
TATGGGTGCTTTTTTTACGTTCTCACGCGTGGCAAGGGGGAGAGCCTCGCCGTGTACAGGGCATCTAA
GCGCCCTTTATTTATTACACAGAGTAAAATAATTCAGTGCCAATTTGTTTGACGGCTATCACGTTTCAA
CTTCAGATCATTGGAGAAGTTGCACATTTTGACAAAACATATGTCACAATATTTCAAACGTGGTTATTC
ACCGCCCAATTTTCGGCGGTGAACTTTACTCGTTTTAGCCATTAATTACGAGCTTTAAAAAAACTCATT
CAACGGATGGATTGAAGGCGGGAAGCGTCGATGTGAAACAGACGGGCGAAGTTATCGGTGGTTACCTGC
GCCAGTCTCGAGAAAAAGGCCATCCGTCAGGATGGCCTTCTGGAGCTC**CTGATGGGGCCGATAGTGTTC**
CTTGCCTCCAGCGCTTCAGATTATGTGAATGGTTATACCATTGCCGTGGATGGCGGTTGGCTGGCGCGT
TAATTCATTCTTCTTACTTTTATGACCCTGCCGCATGGCAGGGTTTTTTATACCTGTAGATCATCATAA
TCCATATCATGGTTATGAAATAATCCATATTAATTATCAATTAATGAACTTTATGAATTTTATCTGCTG
TAAAATTAGGTGGTTAATAATAATCTCAATAATTCAACTTAATTTGAAAATTGGAATATCCATCACATA
ACGACATGTCGCAGCAATTTAATCCATATTTATGCTGTTTCCGACCTGACACCTGCGTGAGTTGTTCAC
GTATTTTTTCACTATGTCTTACTCTCTGCTGGCAGGAAAAAATGGTTACTATCAATACGGAATCTGCTT
TAACGCCACGTTCTTTGCGGGATACGCGGCGTATGAATATGTTTGTTTCGGTAGCTGCTGCGGCTAGCT
TTAAGAAGGAGATATACATATGGTGAGCAAGGGCGAGGAGGATAACATGGCCATCATCAAGGAGTTCAT
GCGCTTCAAGGTGCACATGGAGGGCTCCGTGAACGGCCACGAGTTCGAGATCGAGGGCGAGGGCGAGGG
CCGCCCCTACGAGGGCACCCAGACCGCCAAGCTGAAGGTGACCAAGGGTGGCCCCCTGCCCTTCGCCTG
GGACATCCTGTCCCCTCAGTTCATGTACGGCTCCAAGGCCTACGTGAAGCACCCCGCCGACATCCCCGA
CTACTTGAAGCTGTCCTTCCCCGAGGGCTTCAAGTGGGAGCGCGTGATGAACTTCGAGGACGGCGGCGT
GGTGACCGTGACCCAGGACTCCTCCCTGCAGGACGGCGAGTTCATCTACAAGGTGAAGCTGCGCGGCAC
CAACTTCCCCTCCGACGGCCCCGTAATGCAGAAGAAGACCATGGGCTGGGAGGCCTCCTCCGAGCGGAT
GTACCCCGAGGACGGCGCCCTGAAGGGCGAGATCAAGCAGAGGCTGAAGCTGAAGGACGGCGGCCACTA
CGACGCTGAGGTCAAGACCACCTACAAGGCCAAGAAGCCCGTGCAGCTGCCCGGCGCCTACAACGTCAA
CATCAAGTTGGACATCACCTCCCACAACGAGGACTACACCATCGTGGAACAGTACGAACGCGCCGAGGG
CCGCCACTCCACCGGCGGCATGGACGAGCTGTACAAGTAA

promoter sequences
mCherry
gfp
unique restriction sites
ribosome-binding site
terminator region rrnB T2
