# Supplementary material for: Cell-to-cell variation and specialization in sugar metabolism in clonal bacterial populations
Source: PLoS Genet. 2017 Dec 18;13(12):e1007122. doi: 10.1371/journal.pgen.1007122 (PMC5773225; doi:10.1371/journal.pgen.1007122)
Supplement: S1 File — (PDF) [file pgen.1007122.s016.pdf]

## Supplementary Methods

### Bacterial strains and plasmids

The dual reporter sequence (S1 Sequence) cloned into the company plasmid, forming pNN111, was comprised as follows: AatII-*mCherry*-RBS-NheI-ParaE-SacI-rrnB_T2-XhoI-PptsG-BamHI-RBS-*gfp*-HindIII. Unique restriction sites allowed for swapping of promoters of the construct, and yielded plasmids pNN112, pNN113, pNN114. The promoter regions as well as the strong ribosome-binding site (RBS) were defined as in the *E. coli* promoter-reporter library [Zaslaver et al., 2006].

The modified CRIM plasmids pAH68-frt-chlor and pAH81-frt-chlor were constructed by replacing the ampicillin resistance gene of pAH68 and pAH81 [Haldimann and Wanner, 2001] with a chloramphenicol resistance gene flanked by FRT sites. The FRT-flanked chloramphenicol marker was PCR-amplified from pKD3 [Datsenko and Wanner, 2000] using the primers Fwd_ClaI 5'- TTA TAA TCG ATT ACA CGT CTT GAG CGA TTG T -3' and Rev_NotI 5'-AGC GGC CGC TTA GCC ATG GTC CAT ATG AA -3', and cloned into pAH68 and pAH81 using ClaI/NotI (the underlined sequences correspond to the restriction sites). The resulting plasmids can be inserted into their respective attachment sites (*att*HK022 for pAH68-frt-chlor and *att*P21 for pAH81-frt-chlor) and allow for subsequent removal of the chloramphenicol resistance marker using the site-specific Flp-recombinase [Cherepanov and Wackernagel, 1995].

The fluorescent reporter constructs were amplified with the primers Fwd_EcoRI_pJ (5'- GTA CCG GAA TTC ACT AAG CAG AAG GCC CCT GAC G -3') and Rev_SalI_pJ (5'- TGA CGC GTC GAC AGG GGT CAG TGT TAC AAC CAA TTA ACC -3') and cloned into pAH68-frt-chlor via EcoRI/SalI sites. The amplified constructs were flanked with additional terminators rpn txn and rrnB1 B2 T1 txn (3305 base-pair product). The constructs were integrated in the *E. coli* MG1655 genome into the phage-attachment site *att*HK022, as described in [Haldimann and Wanner, 2001]. The construct from the plasmid pNN111 was also cloned into the modified CRIM vector pAH81-frt-chlor with the same restriction sites and integrated into the *E. coli* MG1655 phage-attachment site *att*P21 [Haldimann and Wanner, 2001]. Subsequently, the chloramphenicol cassette was removed with pCP20 from all strains [Cherepanov and Wackernagel, 1995] and the single-copy states of the insertions verified with the primer sets listed in [Haldimann and Wanner, 2001].

We used *Escherichia coli* K-12 MG1655 harboring a plasmid with promoter-green fluorescent protein (GFP) reporter system for gene expression [Zaslaver et al., 2006] in S8, S9 and S10 Figs. GFP fluorescence thus served as an indirect measurement of a first step in gene expression. We used the strains containing reporters for expression of arabinose uptake systems and metabolism (*araE*, *araF*, *araB*, *araC*), glucose uptake systems (*ptsG*, *mglB*, *manX, malX*), ribosomal protein S13 (*rpsM*), and the promoterless strain MG1655 harboring the plasmid pUA66 without a promoter fused to GFP. The frozen strains of interest were first streaked on LB agar plates containing 50 µg/ml of kanamycin to obtain single colonies. A single colony was then used to inoculate precultures.

**Fixation of bacterial samples**

All chemostat samples were harvested from the liquid phase without shaking or vortexing the glass chemostat-vial. All bacterial samples (batch and chemostat) were first centrifuged at 4000 g for 10 min at 4°C, and re-suspended in 1 ml of 1x PBS. The cells were fixed with 1% formaldehyde in 1x PBS, incubated for 2 hours using a nutator in dark at room temperature, followed by washing with PBS. Finally, the cells were re-suspended in 0.5 ml of 1x PBS and stained with 2 µg/ml Hoechst DNA stain. Of note, sample preparation can affect isotopic enrichment of labeled cells: as reported in [Musat et al., 2014], formaldehyde fixation can lower the $^{13}C$ isotopic enrichment by 3.8 % on average.

**Maximal incorporation of the stable isotope-labeled sugars in chemostats**

In order to obtain information regarding maximal incorporation of $^{2}H$ and $^{13}C$ into bacterial biomass, we did the following experiments under conditions of carbon limitation. After completing five volume changes in mini-chemostats, media-flow was switched to media bottle containing either 20 µM $^{2}H$-labeled glucose or 20 µM $^{13}C$-labeled arabinose; an additional experiment to quantify maximal incorporation of $^{13}C$ into bacterial biomass was switching to media bottle containing 10 µM $^{13}C$-labeled arabinose and 10 µM $^{13}C$-labeled glucose (D-glucose-$^{13}C_6$, 99% labeling, Sigma). The chemostats were run for additional 46 hours (6.9 volume turnovers, corresponding to 9.96 generations) and then harvested. The incorporation of the isotopes into biomass is cumulative and after almost 7 volume changes more than 99% of the biomass should be labeled. This was computed according to the formula: remaining unlabeled biomass= $c/c_0$= exp(-$D*t$), with $D$ as the dilution rate and $t$ as the incubation period.

**AOC contamination in the chemostat setup**

We performed isotope enrichment experiments with $^{13}C$-labeled sugar(s) supplied for ten generations, as described in the previous section. These experiments showed that contaminating AOC in our carbon-limited chemostat setup contributes to 30% of total bacterial biomass, corresponding to about 9 µM arabinose-equivalents (i.e. supplemented 20 µM Ara = 70%, and 9 µM AOC = 30%), and thus supports growth of 0.7 x $10^6$ cells/ml (i.e. 30% of 2.4 x $10^6$ cells/ml, which is the population size in chemostats). This is consistent with the results of an additional experiment described in the section below: carrying capacity of the medium without supplemented sugars is 1.6 x $10^6$ CFU/ml. These values are in line with observations reported in previous studies [Shehata and Marr, 1971]. Moreover, recent studies conducted at our laboratories at Eawag have shown that AOC concentrations are around 1 mg/l ([Helbling et al., 2014], and citations therein), corresponding to 6.7 µM Ara. This concentration is in the range of the experimentally determined 9 µM AOC based on the maximum $^{13}C$ enrichment, when bacteria were grown on 20 µM $^{13}C$-arabinose for ten generations. In general, the sources of AOC in a laboratory setup could be culture containers, contamination in medium components, and laboratory air [Hammes and Egli, 2005].

**Bacterial growth on AOC**

We performed an additional experiment to estimate the amount of biomass that can be assimilated from AOC in our system. Four MG1655 replicate cultures were grown overnight in LB medium, shaking at 37°C, and then diluted 1 to 100 into M9 medium supplemented with 0.2% glucose. On the following day, 2 ml of each culture was pelleted, washed with M9 medium that does not contain any supplemented sugar – M9-Ø medium – and resuspended in 2.5 ml of M9-Ø medium. The cultures were grown overnight in glass culture tubes. The cultures were then diluted 1 to 100 into glass culture tubes with M9-Ø medium and grown overnight. For these cultures colony forming units (CFU) were determined immediately after dilution ('day 0') and 1 day later ('day 1'), by taking 4 samples from each tube and preparing 10-fold serial dilutions in 96-well microplates using a multi-channel pipette (10 µl was transferred into 90 µl of M9-Ø medium). From each dilution 5 µl was pipetted onto an LB agar plate. After drying of the drops at room temperature, the plates were incubated at 37°C for 9 to 13 hours. Colonies were counted for the least diluted spot that still had distinguishable colonies. We calculated an increase of 1.6 x $10^6$ CFU/ml between 'day 0' and 'day 1'.

**Sample preparation for NanoSIMS**

Fixed bacterial samples were applied on polycarbonate filters with a pore size of 0.2 µm (GTTP, Millipore) coated with Au/Pd alloy, and stamped out to get a round filter piece with 5 mm in diameter. The filters were put in a desiccator overnight, in dark, and the following day they were washed first very quickly in sterile Milli-Q filtered $H_20$ and then with absolute ethanol to remove the residual stain. In order to preserve the fluorescence of the strains, the filters were placed on glass microscope slides, mounted in anti-fading medium and stored in dark at 4°C until analyzed with fluorescence microscopy. The anti-fading medium contained 3 parts of Citifluor (Citifluor Ltd., London, UK), and 1 part of VectaShield (Vector Laboratories, CA, USA).

**NanoSIMS measurements**

Using fluorescence microscopy we first selected fields with cells on Au/Pd coated polycarbonate filters [Musat et al., 2008] and marked the areas with a laser micro-dissection microscope Zeiss 200M (Scientific Center for Optical and Electron Microscopy (ScopeM) of ETH Zurich). We recorded images of green and red fluorescence, and fluorescence of the DNA stain for the marked fields (see the section 'Fluorescence microscopy' below, and 'Analysis of filters with fluorescence microscopy and NanoSIMS' in Methods). Selected fields of cells were analyzed with a NanoSIMS 50L (Cameca, Gennevilliers Cedex-France) at the Max Planck Institute for Marine Microbiology in Bremen. The filters were first washed with absolute ethanol to remove the residues of the mounting agents and after drying mounted into NanoSIMS 50L. The areas of interest were pre-sputtered with a $Cs^+$ primary ion beam of 150 pA to remove surface contamination, to implant $Cs^+$ ions in the sample and to achieve an approximately stable ion emission rate. A primary $Cs^+$ with a beam current between 1.5 and 2 pA and a beam diameter around 100 nm were rastered across the cells for analysis.

For each individual cell, secondary ion images of $^1H^-$, $^2H^-$, $^{12}C^-$, $^{13}C^-$ and secondary electrons were simultaneously recorded from analysis area of 10 x 10 µm in raster size and an image size of 256 x 256 pixels with a dwell time of 1 ms per pixel. Analysis area of 20 x 20 µm in raster size and an image size of 512 x 512 pixels were applied for overnight measurements. We measured 50 planes for each position and chose each field of view such that silhouettes of single cells can be distinguished. To minimize interferences the instrument was tuned for high mass resolution (around 7000 MRP). As an internal control of NanoSIMS performance, we measured the facility's *E. coli* sample, which is deuterium unlabeled but $^{13}C$-labeled.

**Fluorescence microscopy**

For measuring fluorescence of the strains cultivated in mini-chemostats we acquired phase contrast and fluorescence images by first applying cells on a 1.5% agarose pad using a cavity slide as described in [Bergmiller et al., 2011]. We harvested 5 ml of bacterial culture from mini-chemostats, and concentrated the cells by immediately spinning them down at 4°C. The pad was inoculated with 1 µl of the concentrated bacterial culture, and the slide was then mounted onto an inverted fluorescence microscope (Olympus IX81), equipped with a cooled CCD camera (Olympus XM10), 100x oil objective, and X-Cite 120PV fluorescence lamp (Lumen Dynamics Group Inc., Canada). Fluorescence images were acquired with a lamp intensity of 50% and exposure time of 300 ms for both GFP filter (Chroma U-N41001: BP 460-500 nm, BA 510-560 nm, DM 505 nm) and RFP filter (Olympus U-MSWG2: BP 480-550 nm, BA 590 nm LP, DM 570 nm). DAPI filter (Olympus U-MNUA2: BP 360-370 nm, BA 420-460 nm, DM 400 nm) was used only for the analysis of NanoSIMS filters. (BP: excitation filter; BA: barrier filter; DM: dichromatic mirror)

**Bleed-through estimation**

In order to quantify the fraction of bleed-through from the GFP signal to the mCherry signal, and *vice versa*, we measured fluorescence of single reporter system based on the plasmids pGFP [Refardt et al., 2013] and pRFP. pRFP has the same sequence as pGFP except the *gfp* gene is replaced by the *mCherry* gene. We grew overnight wild-type strain MG1655 harboring one of these plasmids in M9 salts minimal medium containing 1 mM $MgSO_4$ and 0.1 mM $CaCl_2$, and supplemented with 3 mM D-glucose, 3 mM L-arabinose and 100 µg/ml of ampicillin (AppliChem). The overnight cultured were diluted 100-fold and grown for 2 hours with 250 µM IPTG (Promega). The fluorescence was measured with the lamp intensity set on 50% and exposure time of 100 ms for both filter sets.

Red fluorescence bleed-through factor = red fluorescence (pGFP) / green fluorescence (pGFP)

Green fluorescence bleed-through factor = green fluorescence (pRFP) / red fluorescence (pRFP)

Prior of computing bleed-through factors, fluorescence values were corrected for background and autofluorescence. The mean values for each bleed-through factor were used in subsequent analysis.

## Analysis of fluorescence images taken from agarose pads

Recorded images were analyzed with modified Matlab analysis package *Schnitzcells* [Young et al., 2011], and data was extracted with custom-made Matlab scripts. First, cell outline was determined by phase contrast for images taken from agarose pads. After that, red and green fluorescence signals for each cell were extracted, and values reported in the text are mean values of fluorescence for each cell, defined as total fluorescence divided by the cell area [Young et al., 2011].

**Background correction.** Background fluorescence was determined for each image by quantifying the fluorescence in an area containing no cells. This quantity was subtracted from fluorescence values as determined for cells. The fluorescence is presented in arbitrary units (A.U.), meaning that for every recorded image, its background region had fluorescence of 0 A.U. in each fluorescence channel.

**Autofluorescence correction.** We measured autofluorescence of wild-type MG1655 on agarose pads. The mean autofluorescence values for green and red fluorescence were subtracted from values of the cells of reporter strains.

**Bleed-through correction.** Red fluorescence values were corrected for the bleed-through factor, real red fluorescence= red fluorescence – (green fluorescence * factor), for every analyzed cell. Bleed-through to green fluorescence channel was negligible.

**Cell length.** We used segmentation (phase contrast) images of fluorescence microscopy analysis to infer differences in cell length across different conditions, three replicates per each condition. Cell length in carbon-limited chemostats was 2.20 ± 0.025 µm (mean ± standard error of the mean), and in nitrogen-limited chemostats 3.11 ± 0.060 µm, measured for strain NN114 under defined setup of the fluorescence microscopy. Average variation in cell length was comparable between two conditions, CV of 0.28 for carbon-limited chemostats and 0.30 for nitrogen-limited, carbon-excess chemostats.

## Measurements for estimation of model parameters

This section describes how the maximum growth rates and yields on glucose and arabinose presented in Model Table 1 and Model Table 3 were estimated (see Supplementary Information S2 File, 'Mathematical Model').

Populations of strain NN114 were grown overnight at 37°C in M9 medium supplemented with either 0.6 mM Glc or 0.7 mM Ara, corresponding to 0.01% of each sugar. The next day cultures were diluted 20-fold into fresh medium. After 2.5 hours of growth the cultures were washed twice with M9 medium without sugar and then inoculated to an $A_{600}$ of 0.0001 into fresh M9 medium with either 1.1 mM of glucose or 1.3 mM of arabinose, corresponding to 0.02% of each sugar. Individual wells of a 96-well plate were filled with 200 µL of these cultures, and growth was measured in a BioTek Eon Microplate Spectrophotometer. Resulting $A_{600}$ measurements were background subtracted and the maximum growth rate was determined by fitting an exponential line with base *e* to data between $A_{600}$= 0.03 and $A_{600}$= 0.15. The growth yield was determined by quantifying the cell concentration in the

chemostats ($B_0$= 2.4 x $10^6$ cells $ml^{-1}$), by assuming that growth is for 30% on AOC, and that $y_g$= (6/5)*$y_a$.

**Flow cytometry experiments and data visualization**

Precultures were grown overnight in described minimal media supplemented with D-glucose and/or L-arabinose (Sigma-Aldrich) in defined concentrations, and 50 µg/ml of kanamycin at 37°C. Unless indicated otherwise, the overnight cultures were diluted 1 to 100, grown for 2 hours until early exponential phase and then analyzed with flow cytometer PAS-III (Partec, Muenster, Germany) or FACSCalibur (BD Biosciences, California, USA), both equipped with 488 nm excitation laser. We acquired at least 80,000 events for each flow cytometry measurement, at low speed, with information on FSC-H (forward scatter), SSC-H (side scatter) and FL1-H (GFP fluorescence). Raw data were processed using FlowJo software version 8.8.7 (Tree Star, Inc.), and the cells were gated within a range of defined flow cytometry parameters FSC-H and SSC-H. A rectangle gate 6.98-4532 for FSC and SSC data acquired with PAS-III; and 1.98-100 and 5.05-100 for FSC and SSC data acquired with FACSCalibur was used for data analysis in S8 and S9 Figs to eliminate cell aggregates and other presumably non-bacterial material. For analysis in S10 Fig, a gate was formed on 10,000-12,000 events by using the autogating tool in SSC vs. FSC pseudo-color plots in FlowJo.

**Statistical analysis**

The file S1 Dataset includes source data for figures and tables.

Regarding only NanoSIMS data, we analyzed: 251 cells (6 replicates) of NN114 in carbon-limited chemostats; 112 cells (2 replicates) of NN114 in carbon-excess chemostats; 49 cells (1 replicate) of EAEC in carbon-limited chemostats; 93 cells (3 replicates) of NN114 in carbon-excess batch cultures.

Regarding only fluorescence microscopy, we analyzed: 588 cells (3 replicates from different overnight precultures) of NN114 in carbon-limited chemostats; 240 cells (3 replicates) of NN114 in carbon-excess chemostats; 1917 cells (3 replicates) of NN114 in chemostats with solely arabinose; 2651 cells (3 replicates) of NN114 in chemostats with solely glucose; 888 cells (2 replicates) of NN111 in carbon-limited chemostats; 1086 cells (2 replicates) of NN112 in carbon-limited chemostats; 793 cells (4 replicates) of NN113 in carbon-limited chemostats; 402 cells (2 replicates) of NN111-81 in carbon-limited chemostats.

Lastly, we analyzed 57 cells of NN114 (3 replicates) in carbon-limited chemostats and 81 cells of NN114 (3 replicates) in carbon-excess batch cultures that had information regarding fluorescent transcriptional reporters together with NanoSIMS data.

All statistical tests were performed in SPSS statistical software, version 19. We used Spearman (non-parametric) and Pearson (parametric) two-tailed tests of significance to calculate correlations between datasets. To assess if datasets are drawn from the same

distribution and have the same medians, we used non-parametric tests: Kolmogorov-Smirnov test and Mann-Whitney U test for two independent datasets, and Kruskal-Wallis test for more than two independent datasets. To quantify variation in the sugar assimilation and generation time, we used coefficient of variation (CV), i.e. standard deviation divided by the mean.

## Supplementary References


Bergmiller T, Pena-Miller R, Boehm A, Ackermann M (2011) Single-cell time-lapse analysis of depletion of the universally conserved essential protein YgjD. *BMC Microbiol* **11**: 118

Cherepanov PP, Wackernagel W (1995) Gene disruption in *Escherichia coli*: TcR and KmR cassettes with the option of Flp-catalyzed excision of the antibiotic-resistance determinant. *Gene* **158**: 9–14

Datsenko KA, Wanner BL (2000) One-step inactivation of chromosomal genes in *Escherichia coli* K-12 using PCR products. *Proc Natl Acad Sci U S A* **97**: 6640–6645

Haldimann A, Wanner BL (2001) Conditional-replication, integration, excision, and retrieval plasmid-host systems for gene structure-function studies of bacteria. *J Bacteriol* **183**: 6384–6393

Hammes FA, Egli T (2005) New method for assimilable organic carbon determination using flow-cytometric enumeration and a natural microbial consortium as inoculum. *Environ Sci Technol* **39**: 3289–3294

Helbling DE, Hammes F, Egli T, Kohler HP (2014) Kinetics and yields of pesticide biodegradation at low substrate concentrations and under conditions restricting assimilable organic carbon. *Appl Environ Microbiol* **80**: 1306–1313

Musat N, Halm H, Winterholler B, Hoppe P, Peduzzi S, Hillion F, *et al.* (2008) A single-cell view on the ecophysiology of anaerobic phototrophic bacteria. *Proc Natl Acad Sci U S A* **105**: 17861–17866

Musat N, Stryhanyuk H, Bombach P, Adrian L, Audinot JN, Richnow HH (2014) The effect of FISH and CARD-FISH on the isotopic composition of $^{13}$C- and $^{15}$N-labeled *Pseudomonas putida* cells measured by nanoSIMS. *Syst Appl Microbiol* **37**: 267–276

Refardt D, Bergmiller T, Kümmerli R (2013) Altruism can evolve when relatedness is low: Evidence from bacteria committing suicide upon phage infection. *P Roy Soc B-Biol Sci* **280**: 20123035

Shehata TE, Marr AG (1971) Effect of nutrient concentration on the growth of *Escherichia coli*. *J Bacteriol* **107**: 210–216

Young JW, Locke JC, Altinok A, Rosenfeld N, Bacarian T, *et al.* (2011) Measuring single-cell gene expression dynamics in bacteria using fluorescence time-lapse microscopy. *Nat Protoc* **7**: 80–88

Zaslaver A, Kaplan S, Bren A, Jinich A, Mayo A, *et al.* (2009) Invariant distribution of promoter activities in *Escherichia coli*. *PLOS Comput Biol* **5**: e1000545
