# Supplementary material for: Cell-to-cell variation and specialization in sugar metabolism in clonal bacterial populations
Source: PLoS Genet. 2017 Dec 18;13(12):e1007122. doi: 10.1371/journal.pgen.1007122 (PMC5773225; doi:10.1371/journal.pgen.1007122)
Supplement: S2 File — (PDF) [file pgen.1007122.s017.pdf]

Mathematical Model

Estimates of ^{13}C-arabinose and ^{2}H-glucose uptake from the fractions of heavy isotopes measured in single cells

1. Growth in carbon-limited chemostats

Bacterial populations were grown in minimal media supplemented with 10 µM of glucose and 10 µM of arabinose in carbon-limited chemostats. The media was switched to minimal media containing the same amount of ^{13}C-arabinose and ^{2}H-glucose (referred to as labeled media); after three hours of growth the fraction of labeled atoms in the cell biomass was measured. Based on the measurements, we calculated $X^{E}(^{13}C)_{cell}$ and $X^{E}(^{2}H)_{cell}$, which are the corrected excess atom fractions of ^{13}C and ^{2}H, respectively (see Methods, 'NanoSIMS data analysis'), to estimate the ratio of arabinose over glucose that every cell has taken up. In the following text, the excess fractions $X^{E}(^{13}C)_{cell}$ and $X^{E}(^{2}H)_{cell}$ are denoted as $^{13}C_{ara}$ and $^{2}H_{glc}$, respectively.

$$^{13}C_{ara} = X^{E}(^{13}C)_{cell} \tag{1}$$

$$^{2}H_{glc} = X^{E}(^{2}H)_{cell} \tag{2}$$

After three hours of growth in the labeled media, every cell builds a certain fraction of new biomass and the rest remains old biomass, denoted as C_{old} and H_{old}. The newly assimilated carbon and hydrogen in the cell can originate from at least four sources: water, arabinose, glucose and assimilable organic carbon (AOC). Therefore, the ^{12}C content of the cell originates from three different sources: there is a fraction of carbon from the old biomass, C_{old}, a fraction of carbon that originates from glucose, C_{glc}, and a fraction of AOC, C_{aoc}. Some AOC is always present in the feed medium and the uptake of these compounds makes up a significant fraction of the new biomass when cells are grown under carbon limitation and at low cell densities. Similarly, the ^{1}H content originates from three different sources: there is a fraction of hydrogen from the old biomass, H_{old}, a fraction of hydrogen assimilated together with arabinose, H_{ara}, and fractions of hydrogen assimilated from AOC or exchanged with water that we call $H_{aoc+water}$. From the measured ratios $^{13}C_{ara}$ and $^{2}H_{glc}$ in each cell, we aim to estimate the ratio of arabinose over glucose that the cell has taken up. Therefore, we need to estimate the ratio $^{13}C_{ara}/C_{glc}$. In order to calculate this ratio, we need to solve a system with eight unknowns and eight equations, which we now work out and discuss one by one.

We normalize the contributions of carbon and hydrogen assimilated into the biomass to a total of 1 (Fig 1A), resulting in the two following equations:

$$^{13}C_{ara} + C_{glc} + C_{aoc} + C_{old} = 1 \tag{3}$$

$$^{2}H_{glc} + H_{ara} + H_{aoc+water} + H_{old} = 1 \tag{4}$$

We assume that the fraction of old carbon in the cell is the same as the fraction of old hydrogen in the cell. This corresponds to assuming that the composition in carbon and hydrogen of the biomass is constant during the cell growth throughout the experiment, and that the turnover of atoms in the biomass is similar for carbon and hydrogen atoms. With these assumptions we can write the following equation:

$$C_{old} = H_{old} \qquad (5)$$

The chemical composition of glucose ($C_6H_{12}O_6$) and arabinose ($C_5H_{10}O_5$) suggests the following equation:

$$\frac{^{13}C_{ara}}{H_{ara}} = \frac{C_{glc}}{^{2}H_{glc}} \qquad (6)$$

Moreover, we experimentally estimated the uptake of AOC and of hydrogen from water by growing chemostats with only ^{2}H-glucose or ^{13}C-arabinose (see Supplementary Methods, 'Maximal incorporation of the stable isotope-labeled sugars in chemostats'). The results showed that cells had an excess ^{13}C fraction of 0.707 and an excess ^{2}H fraction of 0.138 despite 10 generations of growth on fully labeled sugars. This means that on average the carbon in the biomass is based on 30% AOC, and the hydrogen in the biomass is based on 86% hydrogen from AOC and water exchange. This results in the following equations:

$$\frac{C_{aoc}}{(1 - C_{old})} = 0.30 \qquad (7)$$

$$\frac{H_{aoc+water}}{(1 - H_{old})} = 0.86 \qquad (8)$$

As we cannot measure these fractions for each single cell in our experimental conditions, we use the equations under the assumption that every cell incorporates the same amount of AOC and exchanges the same amount of water. We have now a system of eight equations and eight variables.

It is important to note that this model is valid if the switch from unlabeled to labeled sugars in the chemostat is instantaneous. However, after the switch there is a certain fraction of ^{12}C-arabinose and ^{1}H-glucose in the chemostat, which decreases through time because of dilution and consumption by cells. The cells grow both on labeled and unlabeled sugars. Therefore, the pool of carbon and hydrogen contains two new terms $^{12}C_{ara}$ and $^{1}H_{glc}$:

$$^{13}C_{ara} + ^{12}C_{ara} + C_{glc} + C_{aoc} + C_{old} = 1$$

$$^{1}H_{glc} + ^{2}H_{glc} + H_{ara} + H_{aoc+water} + H_{old} = 1$$

We denote the total fraction of carbon from arabinose (labeled and unlabeled) C_{ara}, and the total fraction of hydrogen H_{glc}:

$$C_{ara} = ^{13}C_{ara} + ^{12}C_{ara}$$

$$H_{glc} = ^{2}H_{glc} + ^{1}H_{glc}$$

The fraction of labeled atoms that we measured in the cell underestimates the real sugar uptake. Thus, we corrected our estimate of sugar uptake by evaluating how much of the unlabeled sugars the cells take up in addition to the labeled sugars. To this end, we derived the fraction of the unlabeled sugars in the chemostat as a function of time. The average amount of unlabeled sugar in the chemostat during the labeling period of three hours can be found by integrating the concentration of unlabeled sugar over time and by dividing this by the length of time period. We set the switch from the unlabeled to the labeled sugars at time *t*=0, and denote the concentration of unlabeled glucose and arabinose present in the chemostat at time *t* as $G_{unlab}(t)$ and $A_{unlab}(t)$. Then we can write the following differential equations:

$$\frac{d}{dt}G_{unlab}(t) = -DG_{unlab}(t) - \frac{B_0 \mu_g}{y_g}\frac{G_{unlab}(t)}{G_0}$$
$$\frac{d}{dt}A_{unlab}(t) = -DA_{unlab}(t) - \frac{B_0 \mu_a}{y_a}\frac{A_{unlab}(t)}{A_0} \quad (9)$$

The first term represents loss due to dilution (with dilution rate *D*) and the second term represents loss due to consumption; the rate of consumption of unlabeled glucose is the rate of consumption of glucose (labeled and unlabeled) $\frac{B_0 \mu_g}{y_g}$ multiplied by the fraction of unlabeled glucose present in the chemostat $\frac{G_{unlab}(t)}{G_0}$. μ_g and μ_a are the growth rates on glucose and arabinose, respectively, G_0 and A_0 are the standing concentrations of the two sugars in the chemostat which are constant through time, B_0 is the bacterial density in the chemostat, and y_g and y_a are the yields. Refer to Model Table 1 for the values used for these parameters.

Model Table 1. Growth parameters in carbon-limited chemostats supplemented with 10 µM glucose and 10 µM arabinose.

Symbol	Description	Value	Source
D	Dilution rate	0.15 h^{-1}	Fixed in experiment
y_g	Yield on glucose	9.2 x 10^7 cells/µmol	Measured*
y_a	Yield on arabinose	7.6 x 10^7 cells/µmol	Measured*
B_0	Cell density in chemostat	2.4 x 10^6 cells/ml	Measured*
$\mu_{g,max}$	Maximum growth rate on glucose	0.70 h^{-1}	Measured*
$\mu_{a,max}$	Maximum growth rate on arabinose	0.56 h^{-1}	Measured*
K_g	Half-velocity constant for glucose	3.6 µM	Wick et al., 2002**
K_a	Half-velocity constant for arabinose	6.1 µM	Daruwalla et al., 1981***

* Refer to Supplementary Methods for a detailed description of how parameters were measured.

** Estimated from Fig 4 in [Wick LM, Weilenmann H, Egli T (2002) The apparent clock-like evolution of *Escherichia coli* in glucose-limited chemostats is reproducible at large but not at small population sizes and can be explained with Monod kinetics. *Microbiol* **148**: 2889–2902]

*** Taken from Table 8 (CW2004; measured with an *araE* deletion strain) [Daruwalla KR, Paxton AT, Henderson PJF (1981) Energization of the transport systems for arabinose and comparison with galactose transport in *Escherichia coli*. *Biochem J* **200**: 611–627]

Knowing the two initial conditions $G_{unlab}(t) = G_0$ and $A_{unlab}(t) = A_0$, equations (9) can be solved:

$$G_{unlab}(t) = G_0\, e^{-(D+\frac{B_0 \mu_g}{y_g G_0})t}$$
$$A_{unlab}(t) = A_0\, e^{-(D+\frac{B_0 \mu_a}{y_a A_0})t} \qquad (10)$$

The fraction of unlabeled sugars present in the chemostat at time *t* is:

$$\frac{G_{unlab}(t)}{G_0} = e^{-(D+\frac{B_0 \mu_g}{y_g G_0})t}$$

$$\frac{A_{unlab}(t)}{A_0} = e^{-(D+\frac{B_0 \mu_a}{y_a A_0})t}$$

Using these formulae, we can calculate the average fraction of unlabeled glucose and arabinose, g_{unlab} and a_{unlab} , to which the cells have been exposed during three hours, i.e. we calculate the integrals:

$$g_{unlab} = \frac{\int_0^3 G_{unlab}(t)dt}{\int_0^3 G_0 dt}$$
$$a_{unlab} = \frac{\int_0^3 A_{unlab}(t)dt}{\int_0^3 A_0 dt} \qquad (11)$$

To solve equations (11) we need to know the standing concentrations G_0 and A_0 of glucose and arabinose in the chemostat; G_0 and A_0 have not been directly measured, but they can be estimated as follows. The cells in the chemostat follow Monod growth kinetics model, with:

$$\mu_g = \mu_{g,max} \frac{G_0}{G_0 + K_g}$$

$$\mu_a = \mu_{a,max} \frac{A_0}{A_0 + K_a}$$

By inverting these formulae we obtain an estimate of the concentration of glucose in the chemostat:

$$G_0 = \frac{K_g\, \mu_g}{\mu_{g,max} - \mu_g} \qquad (12)$$

and of arabinose:

$$A_0 = \frac{K_a\, \mu_a}{\mu_{a,max} - \mu_a} \qquad (13)$$

To solve (12) and (13) we need estimates of the average growth rates μ_a and μ_g of the cells, which do not need to be very accurate because the corrections g_{unlab} and a_{unlab} are moderately affected by the growth rates, as it is shown in Model Table 2.

Model Table 2. Estimates of the fractions of unlabeled arabinose and glucose, a_{unlab} and g_{unlab} the cells have taken up on average during three hours of growth on labeled sugars.

μ_a *	a_{unlab}	μ_g *	g_{unlab}
0.050 h^{-1}	0.12	0.050 h^{-1}	0.067
0.075 h^{-1}	0.12	0.075 h^{-1}	0.070
0.100 h^{-1}	0.13	0.100 h^{-1}	0.073
0.150 h^{-1}	0.14	0.150 h^{-1}	0.079

*We estimate the growth rates to be in the range μ_a, $\mu_g \in (0.05, 0.15)$ as the average growth rate in the chemostats is known and equal to the dilution rate D= 0.15 h^{-1}.

We estimate the fractions of unlabeled glucose and arabinose, g_{unlab} and a_{unlab} , to which the cells have been exposed during three hours of growth on labeled sugars to be 12% (μ_a = 0.075 h^{-1}) for arabinose and 7% for glucose (μ_g= 0.075 h^{-1}). As a consequence, 12% (7%) of arabinose (glucose) taken up by the cell during three hours is unlabeled, which can be expressed as:

$$\frac{^{1}H_{glc}}{H_{glc}} = g_{unlab}$$

$$\frac{^{12}C_{ara}}{C_{ara}} = a_{unlab}$$

Inserting these equations into $H_{glc} =^1 H_{glc} +^2 H_{glc}$ and $C_{ara} =^{12} C_{ara} +^{13} C_{ara}$, we obtain:

$$C_{ara} = \frac{1}{(1 - a_{unlab})} \quad ^{13}C_{ara}$$

$$H_{glc} = \frac{1}{(1 - g_{unlab})} \quad ^{2}H_{glc} \qquad (14)$$

These are the fraction of carbon (labeled and unlabeled) in the cell biomass originating from arabinose the cell has taken up during three hours, and the fraction of hydrogen (labeled and unlabeled) originating from glucose that has been taken up during the same period.

The system of equations to solve is then:

$$C_{ara} = \frac{1}{(1 - a_{unlab})} \quad {}^{13}C_{ara}$$

$$H_{glc} = \frac{1}{(1 - g_{unlab})} \quad {}^{2}H_{glc}$$

$$C_{ara} + C_{glc} + C_{aoc} + C_{old} = 1 \qquad (15)$$

$$H_{glc} + H_{ara} + H_{aoc+water} + H_{old} = 1$$

$$\frac{C_{ara}}{H_{ara}} = \frac{C_{glc}}{H_{glc}}$$

$$C_{old} = H_{old}$$

$$\frac{H_{aoc+water}}{(1 - H_{old})} = 0.86$$

$$\frac{C_{aoc}}{(1 - C_{old})} = 0.30$$

By solving this system of equations, we can estimate the ratio of arabinose over glucose that each cell has taken up from the ratio of labeled atoms measured in the cell:

$$\left\{\frac{[{}^{13}C]}{[{}^{13}C] + [{}^{12}C]}, \frac{[{}^{2}H]}{[{}^{2}H] + [{}^{1}H]}\right\}_{cell_i} \rightarrow \left\{\frac{arabinose}{glucose} = \frac{{}^{13}C_{ara}}{C_{glc}} \frac{6}{5}\right\}_{cell_i}$$

The factor 6/5 comes from the ratio of C/H atoms in the chemical composition of arabinose and glucose.

We can now estimate isotopic composition of the cell biomass (pie charts in Fig 1A) by solving system (15), and thereby we can also obtain an estimate for the single-cell growth rates on arabinose, glucose, and AOC. These growth rates are the rates of increase of the biomass on each of the carbon sources, calculated as averages during three hours of growth on labeled sugars. According to this definition, we can write the following equations:

$$C_{glc} = (C_{old}\, e^{\mu_{glc} t} - C_{old})|_{t=3}$$

$$C_{ara} = (C_{old}\, e^{\mu_{ara} t} - C_{old})|_{t=3}$$

$$C_{aoc} = (C_{old}\, e^{\mu_{aoc} t} - C_{old})|_{t=3}$$

Inverting these equations we can estimate the growth rates:

$$\mu_{glc} = \frac{1}{3}\ln\left(\frac{C_{old} + C_{glc}}{C_{old}}\right)$$

$$\mu_{ara} = \frac{1}{3}\ln\left(\frac{C_{old} + C_{ara}}{C_{old}}\right)$$

$$\mu_{aoc} = \frac{1}{3}\ln\left(\frac{C_{old} + C_{aoc}}{C_{old}}\right)$$

These formulae have been used to calculate the single-cell growth rates on each carbon source in carbon-limited chemostats.

The cells grow both on glucose and on arabinose and we can define the preference of every cell towards the two sugars as specialization *s*:

$$specialization = \frac{(glucose\ -\ arabinose)}{(glucose +\ arabinose)} = \frac{(C_{glc}5/6\ -\ C_{ara})}{(C_{glc}5/6\ +\ C_{ara})}$$

A specialization of -1 indicates that the cell grows only on arabinose, while a specialization of 1 indicates that the cell grows only on glucose.

2. Growth in batch cultures

The experimentally determined 9 µM AOC (see Supplementary Methods, 'AOC contamination in the chemostat setup') should not be assimilated in bacterial biomass at high carbon concentrations because glucose, if provided in excess, will be taken up preferentially over AOC under this regime of catabolite repression. In our experiments, cells in nitrogen-limited chemostats and batch cultures are indeed under catabolite repression: when grown on both glucose and arabinose, cells do not take up arabinose, and we measured the fraction of ^{13}C from arabinose in the cells to be close to the natural background of ^{13}C (Fig 4B).

Therefore, in batch culture containing 3 mM glucose and 3 mM arabinose, AOC consumption is negligible as compared to sugar consumption, $C_{aoc}^{batch} \cong 0$. We estimate the fraction of hydrogen pertaining to the H_{water}^{batch} term to be 30% lower than the carbon-limited chemostat as it no longer contains 30% of hydrogen coming from AOC, therefore we write:

$$\frac{H_{water}^{batch}}{(1 - H_{old})} \cong 0.6$$

Also, arabinose consumption is negligible, and we can adapt system (15) to the batch growth conditions as follows:

$$H_{glc} = \frac{1}{(1 - g_{unlab})} \quad {}^{2}H_{glc}$$

$$C_{glc} + C_{old} = 1$$

$$H_{glc} + H_{water}^{batch} + H_{old} = 1$$

$$C_{old} = H_{old}$$

$$\frac{H_{water}^{batch}}{(1 - H_{old})} = 0.6$$

Where $g_{unlab} \approx 0.5$ is the fraction of unlabeled sugars in the batch media used in the experiment (see Methods, 'Incubation with the stable isotope-labeled sugars in batch cultures'). The system can be solved and the growth rate on glucose can be obtained as:

$$\mu_{glc} = \frac{1}{0.6} \ln \left(\frac{C_{old} + C_{glc}}{C_{old}} \right)$$

calculated for 0.6 hours of growth on labeled sugars in batch.

3. Growth in nitrogen-limited (carbon-excess) chemostats

In nitrogen-limited carbon-excess chemostats, the cells are consuming only glucose because of catabolite repression. Therefore, growth on arabinose and AOC is negligible, and we set:

$$C_{aoc}^{saturated} \cong 0 \text{ and } \frac{H_{water}^{saturated}}{(1-H_{old})} \cong 0.6$$

as for the batch growth conditions.

The fraction of unlabeled sugars in the chemostat decreases slowly after the switch to the labeled sugars (Fig S3). We estimate the fraction of ^{1}H-glucose to which cells are exposed during three hours of growth in labeled media: in these conditions, growth is limited by nitrogen and the cells take up only a small fraction of glucose present in the media. Therefore, we assume the concentration of glucose in the chemostat to be approximately the same as the incoming concentration (3 mM). Then we can write the following differential equation:

$$\frac{d}{dt} G_{unlab}(t) = -D G_{unlab}(t) - \frac{B_0 \mu_g}{y_g} \frac{G_{unlab}(t)}{G_0} \cong -D G_{unlab}(t) \qquad (16)$$

The consumption term can be neglected because $\frac{B_0 \mu_g}{y_g G_0}$ = 0.0013 << *D*. The consumption term has been estimated using the values for the parameters reported in Model Table 3.

Model Table 3. Growth parameters in nitrogen-limited chemostats supplemented with 3 mM of glucose and 3 mM arabinose.

Symbol	Description	Value	Source
D	Dilution rate	0.15 h^{-1}	Fixed in experiment
G_0	Glucose concentration in chemostat	3 mM	Estimated
B_0	Cell density in chemostat	$2.4x10^6$ cells/ml	Measured
μ_g	Growth rate on glucose	0.15 h^{-1}	Fixed by D
y_g	Yield on glucose	$9.2x10^7$ cells/µmol	Measured

We solve equation (16) with initial condition G_0 = 3 mM and we obtain:

$$G_{unlab}(t) \cong G_0\, e^{-Dt}$$

The fraction of unlabeled glucose still present in the chemostat three hours after the switch is 36%. The fraction of unlabeled glucose to which the cells have been exposed during three hours is:

$$g_{unlab} = \frac{\int_0^3 G_{unlab}(t)dt}{\int_0^3 G_0 dt}$$

We can use g_{unlab} and equation (11) to correct our estimate of glucose uptake; g_{unlab} is equal to 0.80, which means that 80% of the glucose to which the cells are exposed is unlabeled. Gathering together these equations, we obtain the following system:

$$H_{glc} = \frac{1}{(1 - g_{unlab})} \quad {}^2H_{glc}$$

$$C_{glc} + C_{old} = 1$$

$$H_{glc} + H_{water} + H_{old} = 1$$

$$C_{old} = H_{old}$$

$$\frac{H_{water}^{saturated}}{(1 - H_{old})} = 0.6$$

Solving this system allows us to estimate the growth on glucose of every single cell, using the formula:

$$\mu_{glc} = \frac{1}{3} \ln \left(\frac{C_{old} + C_{glc}}{C_{old}} \right)$$

This is the average growth rate of the cell during three hours of growth on labeled sugars in nitrogen-limited chemostats.
